# Silymarin and Zinc Oxide Nanoparticles Attenuate Experimental Colon Cancer in Rats Through Crosstalk of miR-192 and β-Catenin Pathways

**DOI:** 10.3390/biom16091374

**Published:** 2026-09-21

**Authors:** Eman H. Yousef, Aya Megahed, Alaa Samy, Amgad E. Salem, Jawaher Abdullah Alamoudi, Ahmed Abbas, Eman M. Embaby

**Affiliations:** 1 Department of Pharmacology and Biochemistry (Biochemistry), Faculty of Pharmacy, Horus University-Egypt, New Damietta 34518, Egypt; ehamdy@horus.edu.eg; 2 Department of Clinical Pathology, Faculty of Veterinary Medicine, Mansoura University, Mansoura 35516, Egypt; dr.ayamegahed@mans.edu.eg; 3 Department of Surgery, Anesthesiology and Radiology, Faculty of Veterinary Medicine, Mansoura University, Mansoura 35516, Egypt; alaasamy_vet2006@yahoo.com; 4 Department of Pharmaceutics, Faculty of Pharmacy, Mansoura University, Mansoura 35516, Egypt; amgadsalem@mans.edu.eg; 5 Department of Pharmaceutical Sciences, College of Pharmacy, Princess Nourah Bint Abdulrahman University, P.O. Box 84428, Riyadh 11671, Saudi Arabia; 6 Department of Pharmacognosy, Faculty of Pharmacy, Mansoura University, Mansoura 35516, Egypt; aabbas81@yahoo.com; 7 Department of Physiology, Faculty of Veterinary Medicine, Mansoura University, Mansoura 35516, Egypt; emanmohamed@mans.edu.eg

**Keywords:** colon cancer, Silymarin, zinc oxide, nanoparticles, MicroRNAs, β-catenin

## Abstract

Colon cancer (CC) is a leading cause of cancer-related mortality globally. Although chemotherapy remains the primary treatment, its toxicity and drug resistance highlight the need for alternative therapeutic strategies. Silymarin (Sily), a natural compound, and zinc oxide nanoparticles (ZnO-NPs) have shown potential antitumor activity. This study evaluated Sily and/or ZnO-NPs against DMH-induced CC in rats. Thirty male Sprague-Dawley rats were divided into five groups (*n* = 6): control, CC (DMH 20 mg/kg/16-week S.C.), and three groups receiving Sily (40 mg/kg orally), ZnO-NPs (10 mg/kg IP), or their combination daily for 4 weeks. In comparison to the CC group, Sily or ZnO-NPs significantly decreased CEA levels, restored mucin synthesis, and improved colonic histology, with the combination treatment producing the most pronounced antitumor effects. Treatments attenuated oxidative stress, with Sily restoring SOD and GSH levels, whereas ZnO-NPs primarily reduced lipid peroxidation, as reflected by decreased MDA levels. Mechanistically, the combined therapy decreased inhibitory GSK-3β phosphorylation and β-catenin expression, consistent with restoration of GSK-3β-mediated suppression of β-catenin. The combination also restored PKCα and miR-192 expression and reduced the expression of the proliferation- and metastasis-associated markers MMP-9, c-Myc, and Cyclin-D1. CD133 expression was significantly reduced, suggesting an effect on the cancer stem cell-associated phenotype. Sily and ZnO-NPs demonstrated therapeutic effects against CC, particularly when administered in combination. Their antitumor effects were associated with modulation of miR-192/PKCα and GSK-3β/β-catenin signaling, supporting further investigation of their combined multitargeted potential in CC.

## 1. Introduction

Colon cancer (CC) continues to be one of the most frequently diagnosed cancers and a major cause of cancer-related death globally, making it a serious global health concern [1]. 1,2-dimethylhydrazine (DMH) is a carcinogenic compound that induces colorectal tumors closely resembling human colorectal cancer, particularly in their responses to various tumor-promoting and preventive interventions [2]. Azoxymethane (AOM), a metabolite of DMH, and DMH itself both function as cancer-causing substances that need standard metabolic activation to produce genetically reactive intermediates [3]. Several xenobiotic-metabolizing enzymes are involved in their metabolism, which progresses via a number of N-oxidation and hydroxylation processes before hydroxylating AOM to produce methylazoxymethanol (MAM). A methyldiazonium ion is then produced by the reactive metabolite MAM, which alkylates colonic macromolecules and aids in the development of cancer [4].

Most CC cases arise primarily from the dysregulation of the canonical Wnt signaling pathway [5]. Under normal physiological conditions, in colonic crypts, Wnt signaling controls tissue homeostasis and stem cell development [6]. A key effector of this pathway is β-catenin. When Wnt signaling is not triggered, β-catenin binds to adenomatous polyposis Coli, axis inhibition protein, and glycogen synthase kinase 3β to form a complex in the cytoplasm, where it is phosphorylated and then broken down by proteasomes [7,8,9]. However, mutations affecting β-catenin frequently induce inappropriate stimulation of Wnt signaling, which leads to β-catenin stabilizing and building up in the nucleus. Eighty percent of colorectal tumors had nuclear β-catenin accumulation [10]. This suggests that β-catenin is a crucial target for both preventative and therapeutic applications, as well as for tumor development and subsequent promotion of colorectal carcinogenesis [11,12].

Aberrant microRNA (miRNA) expression profiles are hallmarks of malignancy, where they either act as cancer-causing agents or tumor inhibitors [13]. Among these, miR-192 is particularly significant in the gastrointestinal tract, being highly expressed in the normal intestinal epithelium and playing a pivotal role in maintaining epithelial differentiation [14]. It has been documented that several malignant tumors, such as bladder, stomach, and lung cancers, have aberrant expression of miR-192 [15,16,17]. It was shown that CC cells’ expression of miR-192 was significantly lower than that of normal colon cells, particularly the HCT-116 cell line. Moreover, it has been shown that miR-192 overexpression suppresses HCT-116 cell invasion, migration, and proliferation [18].

Conventional chemotherapeutic treatments often demonstrate limited effectiveness, primarily due to drug resistance’s emergence and the occurrence of severe acute and chronic systemic toxicities [19]. This has stimulated growing interest in safer and more effective strategies for cancer management.

In the quest for effective anticancer agents, natural compounds and nanotechnology-based therapies have garnered considerable attention. Silymarin (Sily), a refined extract of the polyphenolic flavonoid complex present in the seeds of milk thistle (Silybum marianum), is well known for its strong hepatoprotective, anti-inflammatory, and antioxidant qualities [20]. Preclinical studies have extensively demonstrated Sily’s broad spectrum of anticancer activities, encompassing the reduction of angiogenesis, activation of apoptosis, prevention of proliferation, and modulation of various signaling pathways implicated in carcinogenesis, across diverse cancer types, including CC [21,22,23,24]. Its favorable safety profile further positions Sily as a promising candidate for chemoprevention and adjunctive therapy.

Concurrently, the burgeoning field of nanotechnology offers innovative solutions for cancer treatment, with zinc oxide nanoparticles (ZnO-NPs) emerging as particularly promising. ZnO-NPs’ capacity to cause oxidative stress, interfere with mitochondrial function, and initiate apoptosis is thought to be the reason for their preferential cytotoxicity towards cancer cells while sparing healthy cells [25]. The small size and unique physicochemical properties of ZnO-NPs enable enhanced cellular uptake and localized drug delivery, potentially overcoming limitations associated with conventional chemotherapeutic agents [26]. Moreover, zinc performs vital roles in many physiological processes as a necessary trace element, and its lack has been associated with an elevated risk of cancer [27].

Therefore, our research evaluates Sily and ZnO-NPs’ individual and combined anticancer properties against DMH-induced CC in a rat model, focusing on tumor progression, biochemical markers, and histopathological alterations. It is proposed that their effects involve inhibition of the Wnt/β-catenin pathway via miR-192 upregulation, highlighting potential as a novel combination therapy.

## 2. Materials and Methods

### 2.1. In Silico Molecular Docking (MD)

The binding affinity of Sily and ZnO-NPs towards GSK-3β was modeled using AutoDock Vina version 1.2.5 and AutoDock Tools version 1.5.7 according to a previously described method [28]. Firstly, the protein data bank (https://www.rcsb.org/pdb/home/home.do) (accessed on 15 March 2026) was utilized to obtain the GSK-3β crystal arrangement (PDB code: 6Y9S). Then, all water molecules and complexes linked to this protein were eliminated. The ionization and tautomeric states of amino acids were modified by the addition of hydrogen. The ZINC database was utilized to obtain the structure of Sily, which was then optimized chemically by altering bond ordering, incorporating charges, and adding hydrogens. In addition, the structure underwent energy minimization. ZnO-NPs’ crystal structure was drawn using VESTA software and used as the ligand. For ZnO-NPs, alpha = beta = 90, gamma = 120, and the parameters of lattice are a = b = 3.24940 and c = 5.20380. The atomic positions of Zn and O in the unit cell are [0.33333, 0.66667, 0.00000] and [0.33333, 0.66667, 0.38210], respectively [29]. A representative ZnO nanocluster (Zn_12_O_12_) was generated and geometrically minimized using the Auto Optimize module in Avogadro with the Universal Force Field (UFF) [30]. The PDBQT file format was used to transform the protein and ligand structures for AutoDock program compatibility. Molecular docking at the OHK ligand binding site was conducted using AutoDock Vina. For the docking process, the binding site of the co-crystallized ligand was selected, and a sphere was created around the protein 6Y9S (radius 8.213767 with X-, Y-, and Z-axes as 0.282618, 21.209309, and 26.639491). Lastly, the predicted docking poses and interactions within the binding pocket were examined and visualized using UCSF Chimera 1.15 and Discovery Studio Visualizer 21.1.0.20298.

### 2.2. String Database Query Method

After visiting the website (http://string-db.org), we chose a multiple protein query and sequentially entered the proteins we wanted (PKCα, β-catenin, GSK-3β, cyclin D1, c-Myc, MMP-9, and CD133). We then obtained a network view with the default configuration to examine cross-links using a weighted scheme that ranked matched annotated proteins.

### 2.3. Chemicals

1,2-Dimethylhydrazine dihydrochloride (98%) (D161802-10G) was purchased from Sigma-Aldrich Munich, Germany, and Sily was obtained from Sigma, St. Louis, MO, USA. Zinc oxide (bulk powder) was supplied by El Nasr Pharmaceutical Chemicals (Al Khanka, Egypt), whereas sodium hydroxide (NaOH) pellets (Cat. No. 567530) were procured from Sigma Aldrich (St. Louis, MO, USA).

### 2.4. Method of Preparation of ZnO-NPs

A co-precipitation technique was employed for the synthesis of ZnO-NPs [31]. Briefly, an alkaline precipitating agent was added to a ZnO precursor solution, resulting in the formation of zinc hydroxide precipitates through simultaneous nucleation and crystal growth. The precipitates were then collected and converted into ZnO NPs by drying and calcination. Bulk ZnO (2 g) was dissolved in dilute 50 mL hydrochloric acid (1 M) under continuous stirring at 60 °C for 30 min until completely dissolved to form a zinc chloride solution. Sodium hydroxide was then gradually added dropwise at room temperature for 24 h while being magnetically stirred at 500 rpm to precipitate zinc hydroxide. The precipitate was collected by centrifugation (6000 rpm, 10 min) and washed three times with deionized water and ethanol until chloride ions were removed. The prepared material was dried at 80 °C for 12 h, followed by calcination at 400 °C for 4 h to obtain ZnO NPs.

### 2.5. Characterization of ZnO-NPs

Several analytical methods were used to characterize the synthesized ZnO-NPs. Dynamic light scattering (DLS) was used to measure the ZnO-NPs’ hydrodynamic particle size (PS) and polydispersity index (PDI) (Malvern Zetasizer, Malvern, Worcestershire, UK). A UV–Vis spectrophotometer (V-530; JASCO Corporation, Hachioji, Tokyo, Japan) was used to capture the ultraviolet–visible (UV–Vis) absorbance spectra of the produced ZnO-NPs. An FTIR spectrometer (Bruker Optik GmbH, Ettlingen, Germany) was used to acquire the Fourier-transform infrared (FTIR) spectra of the synthesized ZnO-NPs spanning the wavenumber range of 400–4000 cm^−1^ in order to determine the distinctive functional groups and chemical bonds present in the NPs. A standard X-ray diffractometer (SmartLab SE, Rigaku, Tokyo, Japan) using Cu-Kα radiation (λ = 1.54 Å) was used to identify the crystalline form and phase structure of the powdered ZnO-NPs. At a voltage of 45 kV and an operating current of 9 mA, the diffraction patterns were captured throughout a 2θ scanning range of 3–80°. Transmission electron microscopy (TEM; JEM-1400 Plus, JEOL, Tokyo, Japan) was used to analyze the morphology, particle dimensions, and form of the produced ZnO-NPs.

### 2.6. Experimental Design

The ZnO-NP powder was suspended in 0.5% CMC to achieve the desired concentration for administration. The suspension was ultrasonicated for 15–30 min to ensure uniform dispersion and prevent the aggregation of NPs. Fresh suspensions were prepared daily before administration to maintain stability.

Thirty male Sprague-Dawley rats were purchased from VACSERA’s animal house in Dokki, Giza, Egypt. They weighed around 200 ± 50 g. The Horus University Central Research Ethics Committee (Ethical code: PH-2025-004) authorized the experimental methods, which were conducted in compliance with the National Institutes of Health’s (NIH) guidelines for the utilization and handling of lab animals (NIH Publication No. 85-23, updated). The animals were kept in typical laboratory settings throughout the acclimatization period, which included a 12 h light/dark cycle, a regulated temperature of 25 °C ± 2 °C, and a relative humidity of 65% ± 5%.

An a priori power analysis was performed using G*Power software (version 3.1.9.4; Heinrich Heine University Düsseldorf, Düsseldorf, Germany) to determine the required sample size for a one-way ANOVA across the five experimental groups. The calculation was based on an effect size of f = 0.70, an α error probability of 0.05, and a statistical power (1−β) of 0.80. The analysis yielded a required total sample size of 30 animals, corresponding to 6 rats per group, with an actual power of 0.809. This sample size was also consistent with previous studies employing the DMH-induced colorectal carcinogenesis model [32,33].

Following two weeks of adaptation, the animals were allocated to the experimental groups using body-weight-stratified randomization in accordance with the ARRIVE 2.0 guidelines [34,35]. Briefly, all animals were weighed and categorized according to their baseline body weight. The 30 rats were divided into six weight groups, each comprising 5 animals with similar body weights. Within each weight group, animals were assigned temporary random identification numbers and randomly allocated to the five experimental groups, with one animal from each weight group assigned to each experimental group. This procedure yielded five groups of six rats with comparable baseline body-weight distributions. Following allocation, each animal was assigned a permanent identification number. Cage placement was also randomized to minimize potential bias associated with housing location [36]. Investigators performing the biochemical and histopathological assessments and statistical analyses were blinded to the experimental group assignments. The five experimental groups were designated as follows:

- Control group: Administered vehicle (1 mM EDTA-saline subcutaneously, S.C.) once weekly for 16 weeks, followed by vehicles (0.5% CMC, p.o., and saline, i.p.) for 4 weeks according to the same administration schedule used for the treated groups.
- Colon Cancer (CC) group: Received DMH (20 mg/kg B.W.) once weekly S.C. for 16 weeks to establish tumor development followed by vehicles (0.5% CMC, p.o., and saline, i.p.) for 4 weeks according to the same administration schedule used for the treated groups [37].
- Sily group: Received DMH (20 mg/kg B.W) once weekly S.C. for 16 weeks, followed post-tumor establishment by Sily (40 mg/kg) orally daily for 4 weeks (Weeks 17–20) [38].
- ZnO-NP group: Received DMH (20 mg/kg B.W.) once weekly S.C. for 16 weeks, followed post-tumor establishment by ZnO-NPs (10 mg/kg, IP) daily for 4 weeks (Weeks 17–20) [39].
- Combined Sily and ZnO-NP treatment group (Sily/ZnO-NPs): Received DMH (20 mg/kg B.W.) once weekly S.C. for 16 weeks, followed post-tumor establishment by both Sily (40 mg/kg, p.o.) and ZnO-NPs (10 mg/kg, IP) daily for 4 weeks (Weeks 17–20).

At the end of the 16-week DMH induction period and before the initiation of treatment, three DMH-exposed rats were randomly selected and sacrificed. Their colons were excised and subjected to histopathological examination to verify the establishment of DMH-induced colorectal neoplastic lesions. Following histological confirmation, the therapeutic intervention phase was initiated, during which Sily (40 mg/kg, orally), ZnO-NPs (10 mg/kg, i.p.), or their combination was administered daily for 4 weeks.

### 2.7. Handling of Biological Samples

At the end of the 20-week experimental period, rats were weighed and anesthetized with pentobarbital sodium (40 mg/kg, i.p.) [40]. After euthanasia, blood samples were extracted from the eye’s retro-orbital vein. Following clot formation for 30 min, samples were centrifuged at 1198× *g* for 10 min to separate the serum, which was then kept at −80 °C. A longitudinal incision was made after the whole colon, from rectum to cecum, was carefully removed and flushed with PBS. For ELISA and biochemical analysis, a 2 cm long section was taken from each colon, put in PBS, and then frozen. They were then ground into a homogeneous mixture and centrifuged for 10 min at 12,000× *g*. After being extracted, the supernatant was kept at −20 °C. Before the experiment, the material was thawed and then centrifuged once more. For real-time PCR and Western blot analysis, a second 2 cm long piece was cut, put in liquid nitrogen, and kept at −80 °C. The remaining colon tissue was preserved for histological and immunohistochemical analysis in 10% buffered formalin.

### 2.8. Calculation of Tumor Incidence and Multiplicity

Tumor incidence was expressed as the percentage of tumor-bearing rats relative to the total number of rats in each group, whereas tumor multiplicity was calculated as the mean number of tumors per tumor-bearing rat [41].

### 2.9. Evaluation of Serum Carcinoembryonic Antigen Level (CEA) by ELISA

The serum concentration of the carcinoembryonic antigen (CEA) was measured using the ELISA kit supplied by MyBioSource Co. (Catalog No: MBS2512373, San Diego, CA, USA), following the manufacturer’s protocol.

### 2.10. Colon Histopathology Assessment

Colon samples fixed in formalin were sectioned and embedded in paraffin. For hematoxylin and eosin (H&E) staining, sections were deparaffinized with xylene, stained with hematoxylin for 1–2 min, and counterstained with eosin for 30 s to 1 min [42]. Histopathological alterations were assessed using a semi-quantitative scoring system based on five features: mucosal and crypt architecture, epithelial dysplasia, inflammatory cell infiltration, goblet cell depletion, and the presence of neoplastic lesions. Each feature was graded from 0 to 4 according to its severity, resulting in a maximum cumulative score of 20 for each animal [43]. For each rat, ten randomly selected microscopic fields were evaluated at ×400 magnification by a blinded investigator. The scores obtained from these fields were then averaged to generate an individual histopathological score for each animal.

For periodic acid Schiff (PAS) staining, sections underwent deparaffinization, rehydration using a graduated ethanol series to distilled water, oxidation with 0.5% periodic acid for 10–15 min, thorough rinsing, and incubation with Schiff’s reagent for 15–30 min at room temperature [42]. Sections were then washed under running tap water, counterstained with hematoxylin, dehydrated, cleared, and mounted. PAS staining was performed to assess mucin content in the colonic crypt epithelium among the experimental groups [42]. For quantitative mucin analysis, the number of PAS-positive goblet cells was counted in 300 colonic crypts for each rat [44].

### 2.11. Analysis of Colon Expression of miR-192 and PKCα

In order to obtain RNA with high purity and integrity, TRIzol reagent (Invitrogen, Waltham, MA, USA) was used to extract total RNA from tissue samples in accordance with the manufacturer’s instructions. Then, the NanoDrop 2000 (Thermo Scientific, Waltham, MA, USA) was used to determine the concentration and purity of the extracted RNA. Only RNA samples exhibiting acceptable A260/A280 ratios were selected for subsequent analyses. An iScript cDNA Synthesis Kit (Bio-Rad, Hercules, CA, USA) was used to generate complementary DNA (cDNA) from total RNA following the manufacturer’s instructions. Quantitative real-time PCR (qRT-PCR) was carried out using SYBR Green chemistry on an ABI 7500 Real-Time PCR System (Applied Biosystems, Waltham, MA, USA) to assess the expression of protein kinase C alpha (PKCα). Gene-specific primers were designed for PKCα and β-actin (primer sequences are listed in Table 1).

For microRNA analysis, the miScript^®^ II RT Kit (Qiagen, Valencia, CA, USA) was used for the synthesis of cDNA. The miScript^®^ SYBR Green PCR Kit (Qiagen) was utilized to perform qPCR amplification in compliance with the manufacturer’s protocol, utilizing miScript Primer Assays specific for miR-192 and U6. Information from the miRBase database was used for primer design, and the primers were supplied by Qiagen (Hilden, Germany). For expression normalization, β-actin was used as the reference gene for mRNA, whereas U6 served as the endogenous control for miRNA.

The settings for PCR amplification included an initial 10 min enzyme activation stage at 95 °C, 40 cycles of denaturation at 95 °C for 15 s, and annealing/extension at 60 °C for 60 s. To confirm the amplified products’ specificity, a melt curve analysis was carried out. Following normalization to the corresponding endogenous control, relative gene expression levels were computed using the 2^−∆∆ct^ method and displayed as fold changes in relation to the control group [45].

### 2.12. Determination of Cyclin D1 and β-Catenin by ELISA

The concentration of cyclin D1 and β-catenin (cadherin-associated protein beta, 88 kDa) in rat colon tissue was measured using commercial ELISA kits (Cyclin D1: Cat. No. MBS043628; β-catenin: Cat. No. MBS843456) following the manufacturers’ instructions. Colon tissue homogenates were processed, and optical density readings were obtained using an ELISA reader.

### 2.13. Determination of Glycogen Synthase Kinase-3 Beta (GSK3β) (Phosphorylated and Total) by Western Blot Technique

Western blotting was used to assess the protein levels of total glycogen synthase kinase-3β (GSK3β) and phosphorylated GSK3β (p-GSK3β) in colon tissues. Briefly, colon tissue samples were homogenized in lysis buffer, and the homogenates were centrifuged at 18,000× *g* for 1 h at 4 °C. The obtained supernatants were collected for protein analysis. A commercial protein extraction kit was used to isolate total proteins in accordance with the manufacturer’s instructions. Coomassie Brilliant Blue (Bradford) protein assay reagent (Pierce, Rockford, IL, USA) was used to quantify protein concentrations.

For electrophoretic separation, equal amounts of protein (30 μg per sample) were loaded onto 8% sodium dodecyl sulfate–polyacrylamide gels (SDS-PAGE) and subsequently transferred onto nitrocellulose membranes. The membranes were blocked by incubation for 1 h at 22 °C in 5% non-fat dry milk prepared in phosphate-buffered saline containing 0.5% Tween-20 (PBS-T; 137 mM NaCl, 2.7 mM KCl, 10 mM Na_2_HPO_4_, and 2 mM KH_2_PO_4_; pH 7.4) to prevent non-specific binding.

After membrane blocking, the membranes underwent incubation with primary antibodies towards total GSK3β (rabbit polyclonal antibody, 1:1000; Catalogue No. PA5-29251, Invitrogen, Waltham, MA, USA) and phospho-GSK3β (Ser9) (rabbit monoclonal antibody, 1:1000; Catalogue No. MA5-14873, Invitrogen, Waltham, MA, USA). Following the incubation of the primary antibody, membranes were rinsed three times with PBS before being incubated for one hour at room temperature with the goat anti-rabbit IgG secondary antibody (Novus Biologicals) coupled with horseradish peroxidase (HRP). Improved chemiluminescence Western blotting reagents (Bio-Rad, Hercules, CA, USA) were used to identify protein bands, which were then visualized by exposure to X-ray film. ImageJ software (version 1.53s) was used to quantify band intensities, and the housekeeping protein β-actin was used to normalize the expression levels of target proteins.

### 2.14. Expression of c-Myc Protein, MMP-9, and CD133 by Immunohistochemistry

Immunohistochemical analysis of c-Myc, matrix metalloproteinase-9 (MMP-9), and CD133 was carried out on 2–4 μm sections prepared from formalin-fixed, paraffin-embedded (FFPE) colon tissues. Paraffin-embedded tissue sections were deparaffinized with xylene and rehydrated using decreasing concentrations of ethanol. The sections were then incubated at 4 °C overnight with primary antibodies targeting c-Myc (Cat. No. ab32072, Abcam, Cambridge, UK), MMP-9 (Cat. No. ab58803, Abcam), and CD133 (Cat. No. ab19898, Abcam). After incubation with the primary antibodies, HRP-conjugated secondary antibodies (Abcam) were applied. Immunostaining was developed with 2% diaminobenzidine (DAB) in 50 mM Tris buffer (pH 7.6), followed by hematoxylin counterstaining. The stained sections were examined by light microscopy by an investigator blinded to group allocation. Digital images were subsequently evaluated using ImageJ, and immunoreactivity was quantified as the percentage of positively stained area.

### 2.15. Assessment of Colon Oxidative Stress and Antioxidant Activities

Using easily accessible Biodiagnostics kits (Cairo, Egypt), oxidative stress and antioxidant markers were assessed using colon homogenate as GSH (Cat.no.GR.2511), MDA (Cat.no.MD 2529), and SOD (Cat.no.SD 2521). The measurements were completed in accordance with the manufacturer’s instructions.

### 2.16. Statistical Analysis

Statistical analyses were conducted using GraphPad Prism 8.0.2 (GraphPad Software, San Diego, CA, USA). Comparisons among experimental groups were performed by one-way ANOVA followed by Tukey’s multiple-comparison test. Six biological samples from each group were included in tissue-based assessments (*n* = 6), whereas Western blot experiments were conducted using three independent biological samples per group (*n* = 3). For total histopathological score, Kruskal–Wallis test followed by Dunn’s multiple-comparison test was performed. For correlation analyses, data distribution was examined using the Shapiro–Wilk test. Since not all variables fulfilled the assumption of normality, relationships between miR-192 expression and the examined molecular parameters were assessed using two-tailed Spearman’s rank correlation (ρ). Results are expressed as the mean ± SEM, and statistical significance was accepted at *p* < 0.05.

## 3. Results

### 3.1. Characterization of ZnO-NPs

#### 3.1.1. Particle Size Analysis

The ZnO-NPs exhibited a mean hydrodynamic PS of 149 ± 2.3 nm, with a PDI of 0.321 ± 0.06, indicating a relatively narrow particle size distribution. The obtained nanoscale particle size confirmed the successful formation of ZnO-NPs. The hydrodynamic diameter measured by DLS was higher than the particle size observed by transmission electron microscopy (TEM), which could be explained by the presence of the hydration layer surrounding the NPs and possible differences in sample dispersion conditions [46].

#### 3.1.2. UV–Vis Analysis

**Figure 1:**
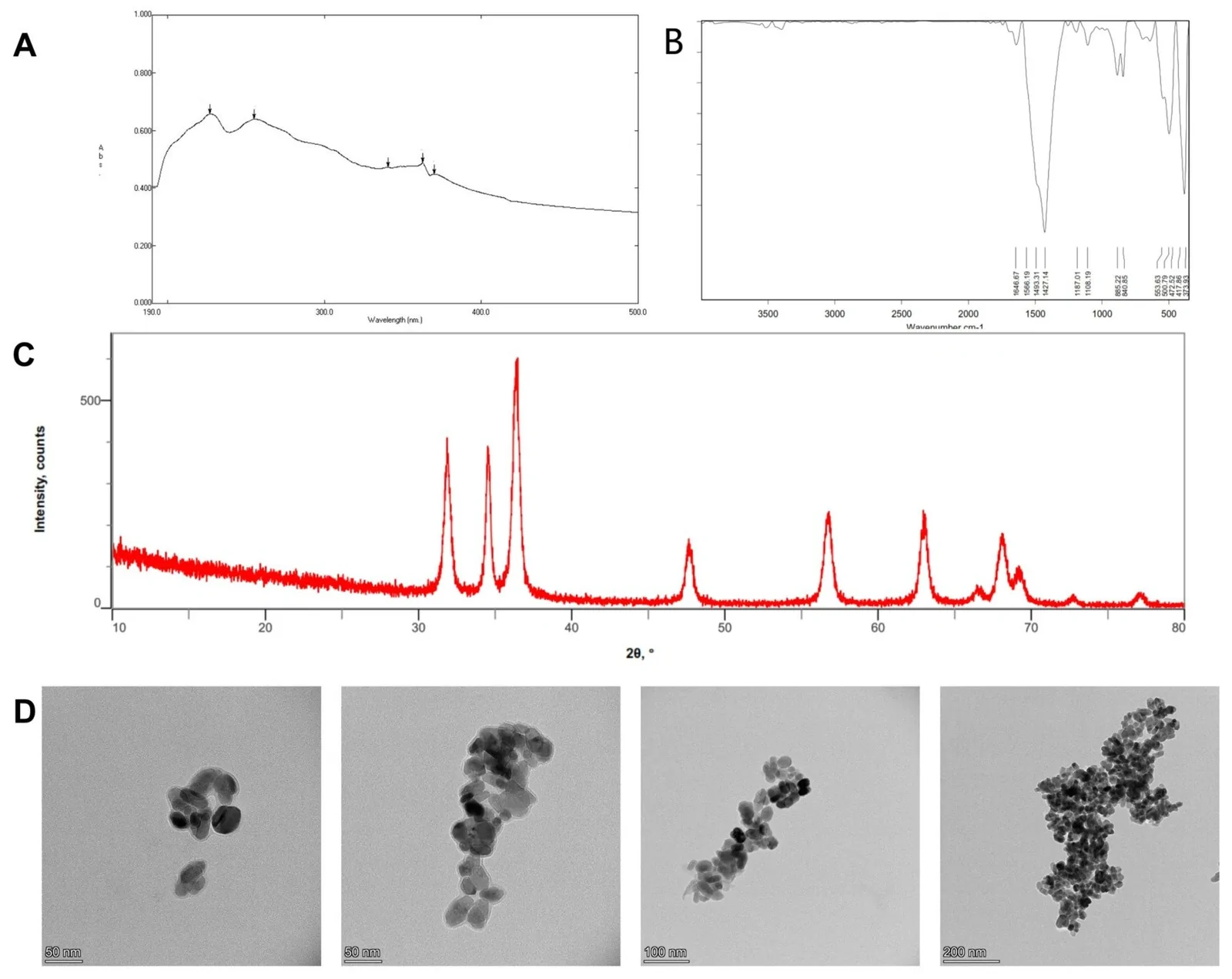
Characterization of the synthesized ZnO-NPs: (**A**) UV–Vis absorption spectrum; (**B**) FTIR spectrum; (**C**) X-ray diffraction (XRD) pattern; and (**D**) transmission electron microscopy (TEM) micrographs.

The UV–Vis spectroscopic analysis of the synthesized ZnO-NPs exhibited a characteristic absorption maximum at approximately 362 nm (Figure 1A). This absorption peak is in agreement with values for ZnO-NPs described in previous studies [47]. In comparison, bulk ZnO typically exhibits an absorption edge at approximately 380 nm. The observed blue shift in the absorption maximum from 380 nm for bulk ZnO to approximately 362 nm for the synthesized ZnO-NPs could be related to the nanoscale dimensions of the particles and the associated quantum confinement effect. This spectral shift provides supportive evidence for the successful ZnO-NP formation.

#### 3.1.3. FT-IR Spectra

The FTIR spectrum of the synthesized ZnO-NPs showed a characteristic absorption band in the lower wavenumber region, approximately 400–600 cm^−1^ (Figure 1B), which can be attributed to the Zn–O bond stretching vibration, supporting the formation of ZnO-NPs [48].

#### 3.1.4. XRD Analysis

X-ray diffraction (XRD) analysis was performed to examine the crystalline structure of the synthesized ZnO-NPs. The diffraction pattern revealed distinct peaks at 2θ values of 31.8°, 34.2°, 36.3°, 47.59°, 56.66°, 62.93°, 66.53°, 68.02°, 69.15°, 72.65°, and 77.04°, which were indexed to the corresponding crystallographic planes (100), (002), (101), (102), (110), (103), (200), (112), (201), (004), and (202), respectively (Figure 1C). The obtained diffraction peaks were consistent with the characteristic hexagonal wurtzite structure of ZnO based on the standard JCPDS card No. 36–1451, confirming the crystalline nature of the prepared NPs [47]. Furthermore, the absence of additional diffraction peaks attributable to impurities or intermediate phases suggests the formation of highly pure ZnO-NPs.

#### 3.1.5. TEM

Figure 1D presents the TEM micrographs of the synthesized ZnO-NPs. The NPs exhibited a predominantly hexagonal morphology. TEM analysis revealed nanosized ZnO primary particles with a tendency to form aggregates/agglomerates. Although individual nanoparticles could be observed, particle aggregation was evident in several fields of view.

### 3.2. Sily and ZnO-NPs Mitigate DMH-Induced CC

Tumor incidence reached 100% in the DMH-induced CC group. In contrast, treatment with Sily, ZnO-NPs, or their combination was associated with a reduction in tumor incidence. Similarly, the increased tumor multiplicity observed following DMH administration was reduced in all treatment groups, with the Sily/ZnO-NPs combination showing an improvement compared with the individual treatments (Table 2).

**Figure 2:**
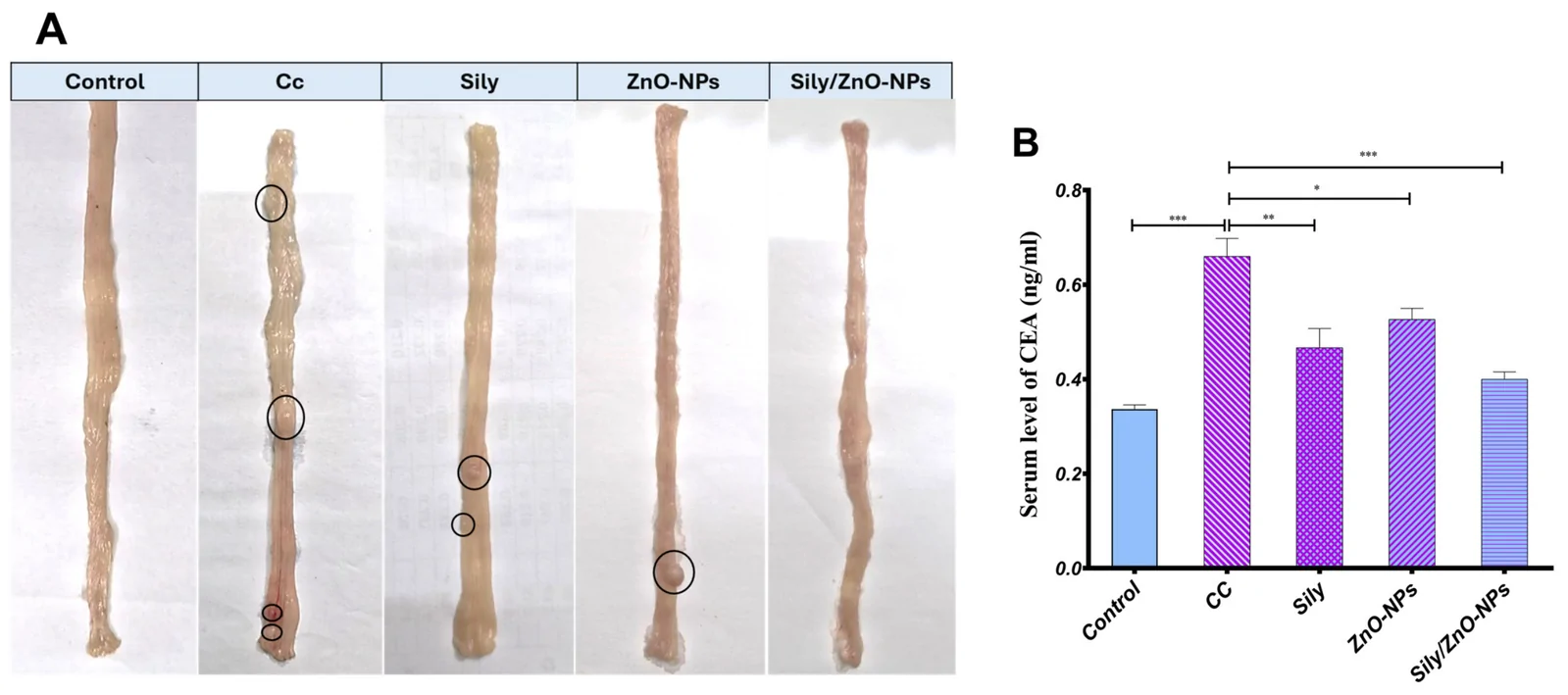
Sily and ZnO-NPs mitigate DMH-induced CC. (**A**) Representative gross appearance of the colon from the different experimental groups; black circles indicate visible tumor nodules. (**B**) Serum carcinoembryonic antigen (CEA) levels (ng/mL) determined by ELISA across the different groups. Data are presented as the mean ± standard error of the mean (SEM). Statistical significance is indicated as follows: * *p* < 0.05, ** *p* ≤ 0.01, and *** *p* ≤ 0.001.

DMH administration at a dose of 20 mg/kg body weight every week for 16 weeks resulted in a marked elevation in serum CEA by 96% (*p* ≤ 0.001) in comparison with the control group. Conversely, administration of Sily, ZnO-NPs, and Sily/ZnO-NPs effectively reduced elevated serum CEA by 29.3% (*p* ≤ 0.01), 20.2% (*p* < 0.05), and 39.4% (*p* ≤ 0.001) relative to the CC group (Figure 2).

**Figure 3:**
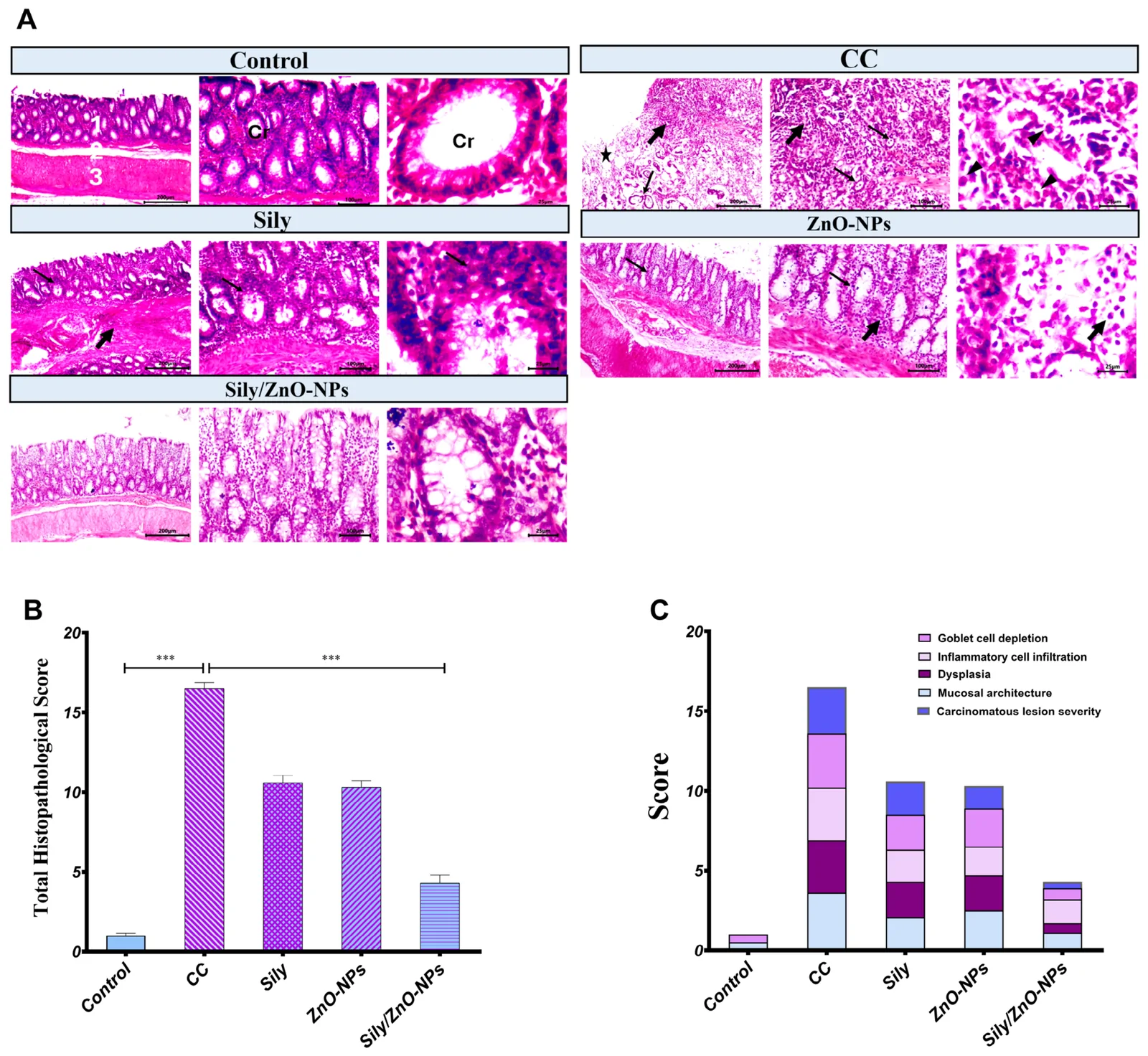
Sily and ZnO-NPs mitigate DMH-induced histopathological alterations. (**A**) Histopathological examination of colon tissue stained by H&E in different groups. Here, 1 represents intact mucosa, 2 represents submucosa, 3 represents muscular layers, Cr represents regularly arranged crypts, thick black arrows represent inflammatory cell infiltration, * represents edema, and black arrowheads represent atypical mitotic activity. Magnification x: 40 scale bar 200 μm, x: 100 scale bar 100 μm, and x: 400 scale bar 25 μm. (**B**) Total histopathological scores of colon tissues from different groups. (**C**) Scores for the evaluated histopathological parameters, including mucosal and crypt architecture, epithelial dysplasia, inflammatory cell infiltration, goblet cell depletion, and neoplastic lesions. Data are presented as the mean ± SEM (*n* = 6). *** *p* ˂ 0.001.

The histopathological study of colon slices from the control colonic wall revealed normal histological findings in the mucosa, submucosa, and muscular layers, as well as normal mucosal cellular structure characterized by a goblet cell-rich epithelium. In contrast, administration of DMH to rats revealed severe distortion of colonic architecture with severe dysplasia (thin arrows), severe inflammatory cell infiltration (thick arrows), edema (star) and marked epithelial atypia characterized by nuclear pleomorphism, hyperchromasia, loss of polarity, and atypical mitotic activity. On the other hand, Sily, ZnO-NPs, and Sily/ZnO-NPs obviously mitigated the histopathological changes induced by DMH. Sily revealed partial improvement, with moderate dysplasia, hyperplastic crypts (thin arrows), and moderate inflammatory cell infiltration (thick arrow). Colonic tissue sections from rats treated with ZnO-NPs revealed greater improvement, with partially preserved colonic architecture, mild dysplastic crypts (thin arrow), and mild inflammatory cell infiltration (thick arrows). Colonic tissue sections from rats treated with Sily/ZnO-NPs revealed near normal colonic architecture, with preserved crypts and goblet cells (Figure 3A).

Histopathological scoring supported these observations. Compared with the control group, CC group exhibited significantly higher total histopathological score by 16.5-fold (*** *p* < 0.001). Notably, Sily/ZnO-NPs significantly reduced the total histopathological score by 3.8-fold (*** *p* < 0.001) relative to the CC group (Figure 3B,C).

### 3.3. Sily and ZnO-NPs Mitigate DMH-Induced Loss of Colon Mucin

**Figure 4:**
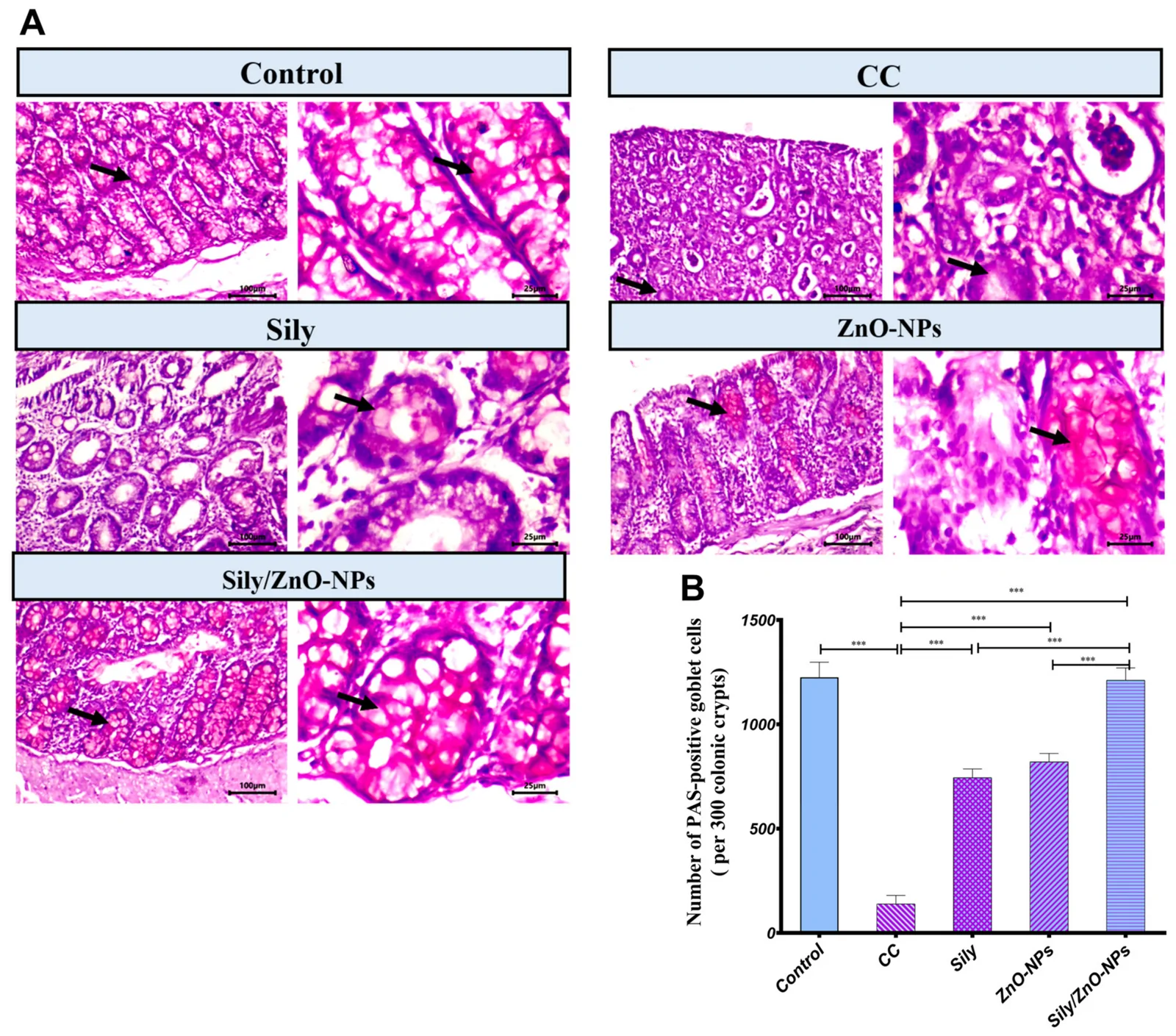
Sily and ZnO-NPs mitigate DMH-induced loss of colon mucin. (**A**) Histopathological examination of colon tissue stained by PAS in different groups. Black arrows represent PAS-positive mucus in the crypt epithelium. Magnifications X: 100 = scale bar 100 μm and X: 400 = scale bar 25 μm. (**B**) Quantitative analysis of PAS-positive goblet cells in colonic tissue. PAS-positive goblet cells were quantified by counting individual PAS-positive cells in 300 evaluable colonic crypts per rat. Data are presented as the mean ± SEM (n = 6 rats/group). Data analyzed by one-way ANOVA followed by Tukey’s multiple-comparison test. (*** *p* ≤ 0.001).

In the control group, PAS-stained colon sections showed normal intestinal glandular architecture and normal goblet cell aberration. In contrast, microscopic pictures of PAS-stained colonic sections showed markedly decreased PAS-positive mucus in the crypt epithelium in the CC group by 88.67% (*p* ≤ 0.001) as compared to the control group. PAS-stained colonic sections from the Sily-, ZnO-NP-, and Sily/ZnO-NP-treated groups showed a significant increase in PAS-positive mucin in the crypt epithelium by 436%, 490%, and 772.3% (*p* ≤ 0.001) as compared to the CC group, respectively. PAS-stained colonic sections from Sily/ZnO-NP-treated group showed a significant increase in PAS-positive mucin in the crypt epithelium by 62.7% and 47.7% (*p* ≤ 0.001) as compared to the Sily and ZnO-NP groups, respectively (Figure 4).

### 3.4. Sily and ZnO-NPs Reverse DMH-Induced miR-192 and PKCα Suppression

**Figure 5:**
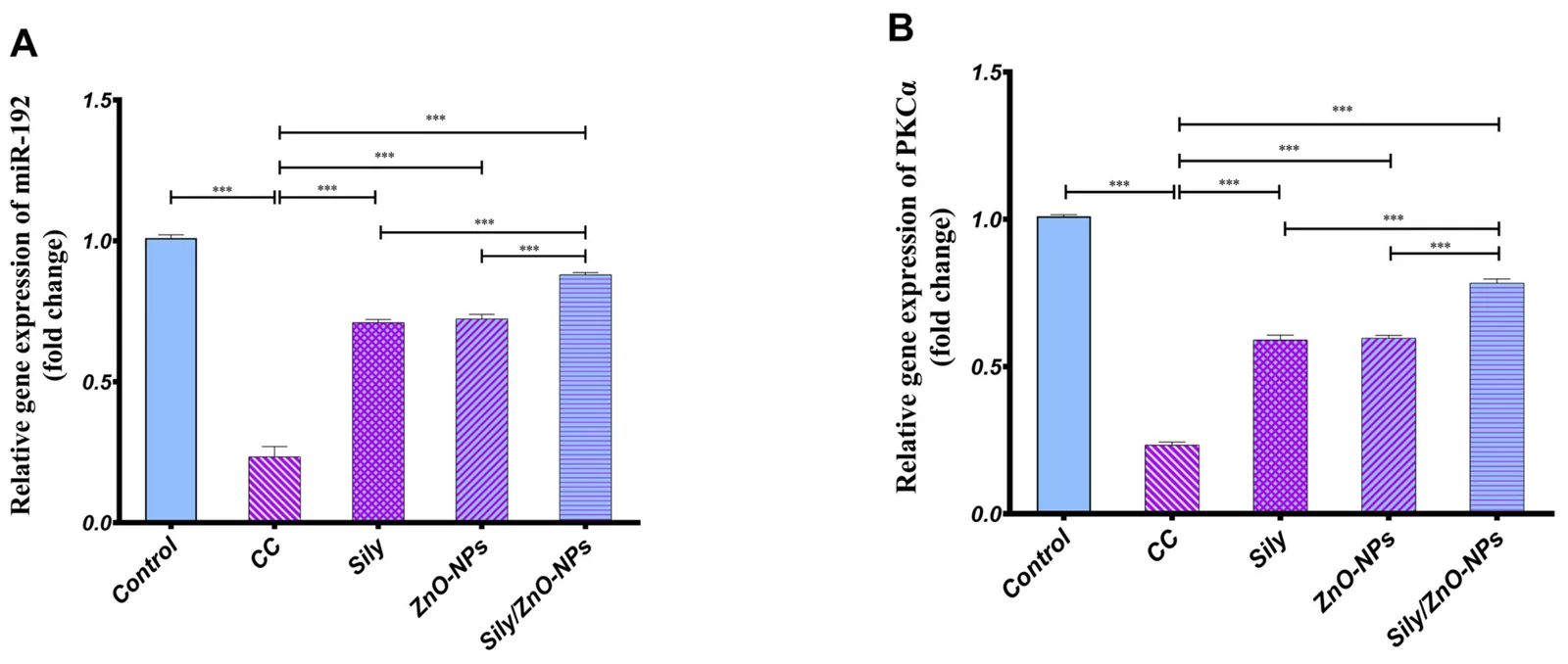
Sily and ZnO-NPs reverse DMH-induced miR-192 and PKCα suppression. Effect of Sily, ZnO-NPs, and Sily/ZnO-NPs on DMH-induced miR-192 (**A**) and PKCα (**B**) suppression was measured across the different experimental groups by qRT-PCR. Data are presented as the mean ± standard error of the mean (SEM). *** *p* ≤ 0.001.

The impact of Sily, ZnO-NPs, and Sily/ZnO-NPs on the relative gene expression levels of miR-192 and PKCα in the colonic tissues from DMH treated rats is illustrated in Figure 5. The miR-192 and PKCα levels in colonic tissues from rats treated with DMH for 16 weeks were significantly lower by 76.9% and 77% (*p* ≤ 0.001) in comparison with the control group, respectively. Nevertheless, colonic tissue from rats treated with Sily, ZnO-NPs, and Sily/ZnO-NPs showed a significant elevation in miR-192 levels by 204%, 210%, and 277%, at *p* ≤ 0.001, respectively, as compared to the CC group. Additionally, treatment of animals with the Sily/ZnO-NP combination resulted in a significant increase in colonic miR-192 compared with the Sily and ZnO-NP groups by 23.9% and 21.7%, at *p* ≤ 0.001, respectively. In addition, the colon tissues of the animals treated with Sily, ZnO-NPs, and Sily/ZnO-NPs displayed a significant elevation in relative mRNA expression of PKCα by 152.9%, 155.7%, and 235.7%, at *p* ≤ 0.001, respectively, in comparison with the CC group. Moreover, treatment of animals with the Sily/ZnO-NP combination resulted in a significant increase in colonic relative mRNA expression of PKCα as compared with the Sily and ZnO-NP groups by 32.8% and 31.3%, at *p* ≤ 0.001, respectively.

### 3.5. Sily and ZnO-NPs Restore GSK-3β-Mediated Regulation of β-Catenin in DMH-Induced CC

**Figure 6:**
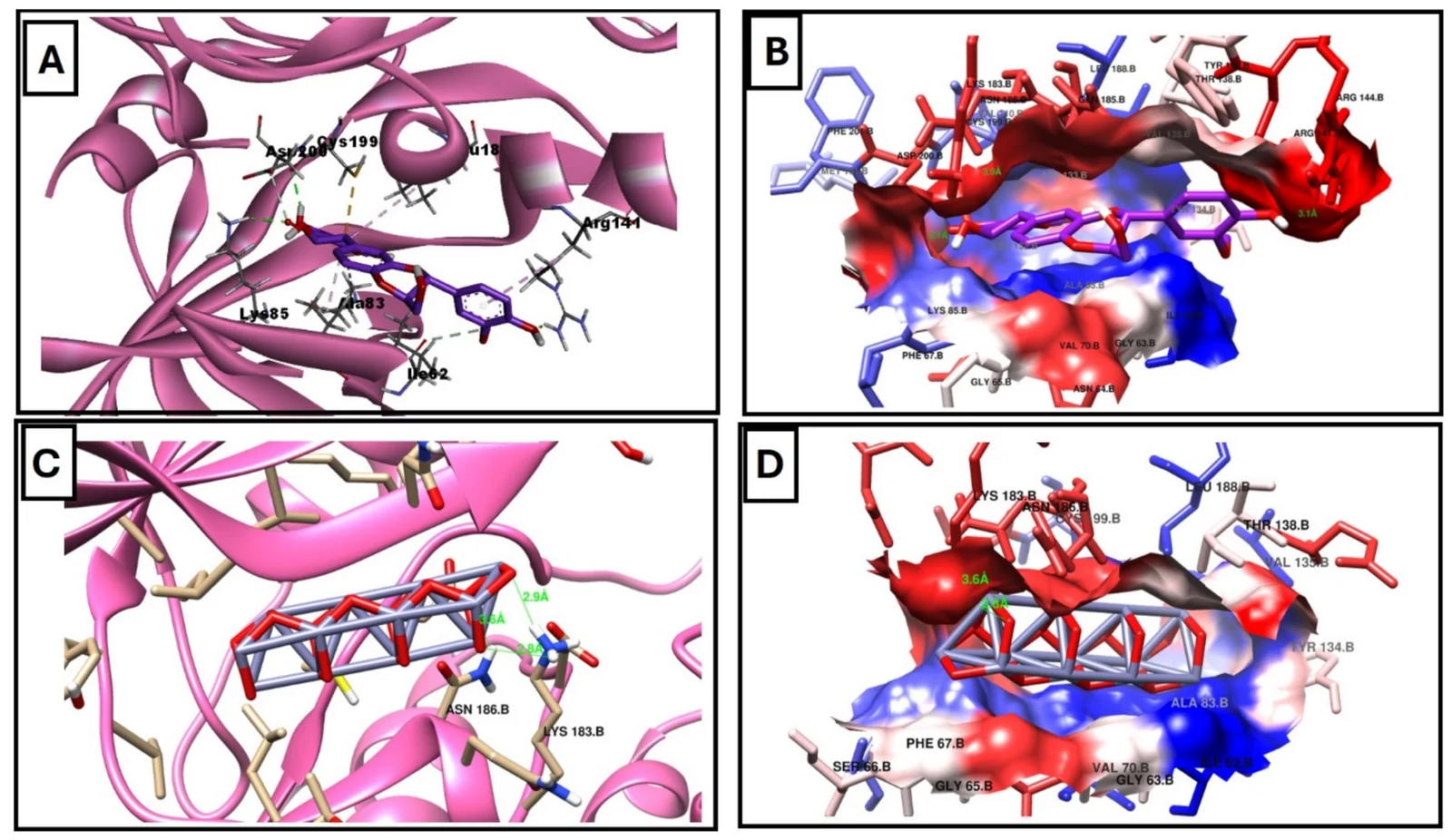
Molecular docking analysis of Sily and ZnO-NPs with GSK-3β. (**A**) Amino acid residues involved in the interaction between GSK-3β (PDB ID: 6Y9S) and Sily, visualized using Discovery Studio. (**B**) Hydrophobic interactions between Sily and the GSK-3β binding pocket, analyzed with Chimera. (**C**) Amino acids participating in the interaction of GSK-3β (PDB ID: 6Y9S) with ZnO-NPs, visualized using Chimera. (**D**) Hydrophobic interactions between ZnO-NPs and the GSK-3β pocket, illustrated using Chimera. The molecular surface is colored according to hydrophilic/lipophilic character, with blue indicating lipophilic regions and red indicating hydrophilic regions.

To evaluate the potential interactions of Sily and ZnO-NPs with GSK-3β, molecular docking analysis was performed using AutoDock Vina 1.1.2 [49]. Sily and ZnO-NPs exhibited predicted binding affinities of −9.5 and −7.8 kcal/mol, respectively, at the OHK binding site of GSK-3β. Sily showed predicted hydrogen-bond interactions with ARG141, LYS85, and CYS199, along with hydrophobic interactions involving ALA83, ILE62, LEU132, VAL70, PHE67, VAL135, LEU188, VAL110, CYS199, PHE201, and MET101. ZnO-NPs showed predicted hydrogen-bond interactions with LYS183 and ASN186, together with hydrophobic interactions involving LEU188, VAL135, ALA83, ILE62, VAL70, PHE67, GLY63, CYS199, GLY65, THR138, SER66, and TYR134. These docking findings suggest potential interactions with GSK-3β and provide supportive in silico evidence that requires experimental validation of direct target engagement. The predicted binding poses and interactions are presented in Figure 6 and Table 3.

**Figure 7:**
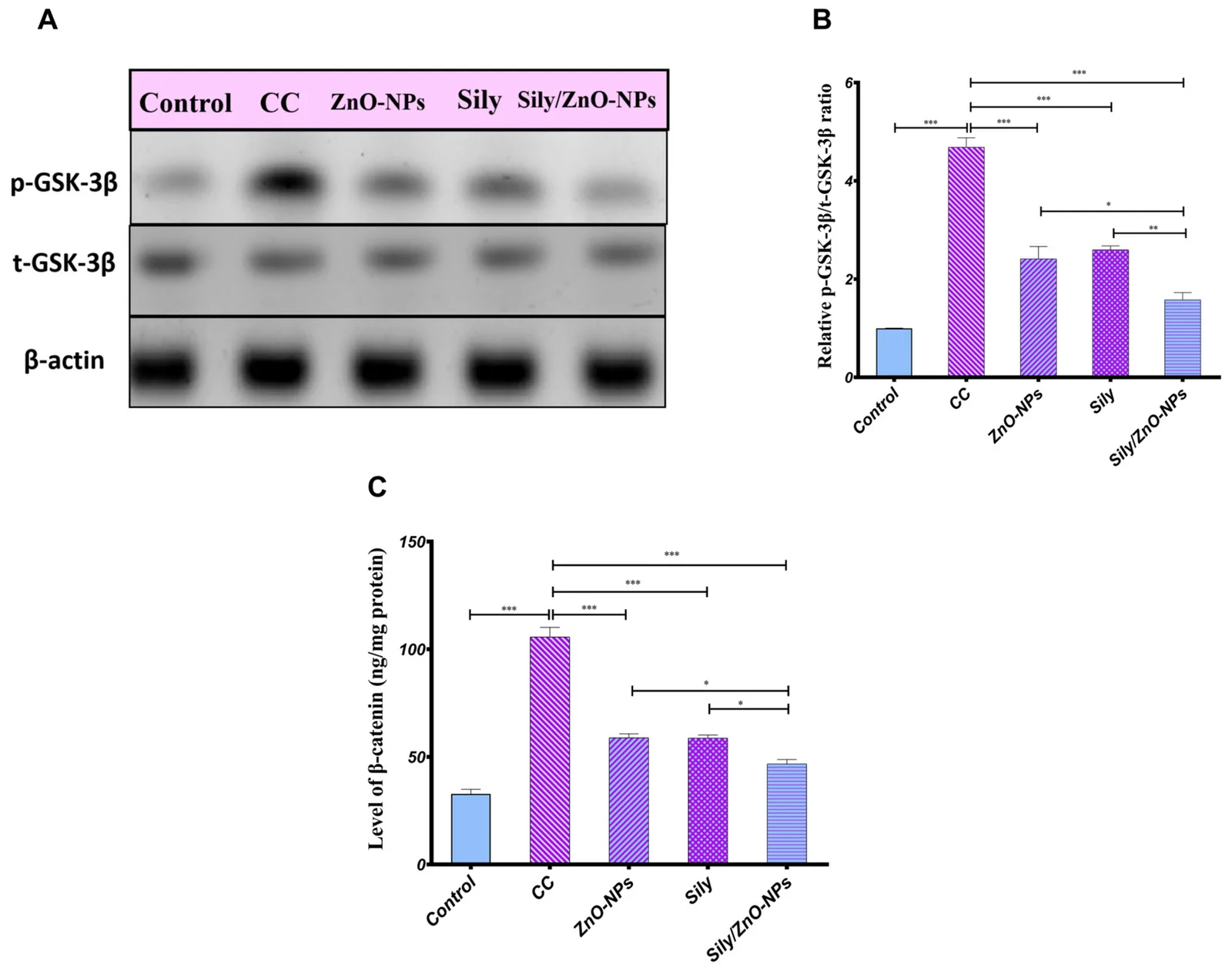
ZnO-NPs and Sily inhibit GSK-3β/β-catenin pathway in DMH-induced CC. (**A**) The impact of ZnO-NPs, Sily, and their combination Sily/ZnO-NPs on t-GSK-3β and p-GSK-3β protein levels was evaluated across the experimental groups using Western blot analysis. The original Western Blot images can be found in Supplementary Materials. (**B**) Quantitative analysis of Western blot. (**C**) Level of β-catenin (ng/mg protein) measured by ELISA in different groups. Data are presented as the mean ± standard error of the mean (SEM). Statistical significance is indicated as follows: * *p* < 0.05, ** *p* ≤ 0.01, and *** *p* ≤ 0.001.

Moreover, the levels of β-catenin and p-GSK-3β were determined. The findings demonstrated that β-catenin and p-GSK-3β levels were remarkably increased by 222.3% and 369.9% (*p* ≤ 0.001) in the CC group relative to the control group, respectively. Conversely, the colonic tissues from the animals treated with Sily, ZnO-NPs, and Sily/ZnO-NPs exhibited a marked decrease in β-catenin levels by 44.4%, 44.3%, and 55.8%, at *p* ≤ 0.001, respectively, compared with the CC group. Also, Sily-, ZnO-NP-, and Sily/ZnO-NP-treated groups showed a significant decrease in *p*-GSK-3β levels by 44.6%, 48.5%, and 66.3%, at *p* ≤ 0.001, respectively, relative to the CC group. Moreover, treatment of animals with the Sily/ZnO-NP combination led to a marked decline in β-catenin and p-GSK-3β levels compared with the Sily group by 20.4% and 39.2%, at *p* < 0.05 and *p* ≤ 0.01, respectively. Also, there was a marked reduction in the β-catenin and p-GSK-3β levels in Sily/ZnO-NP-treated group by 20.6% and 34.6% %, at *p* < 0.05, respectively, in comparison with the ZnO-NP group (Figure 7).

### 3.6. Sily and ZnO-NPs Exert Antiproliferative Activity Against DMH-Induced CC

**Figure 8:**
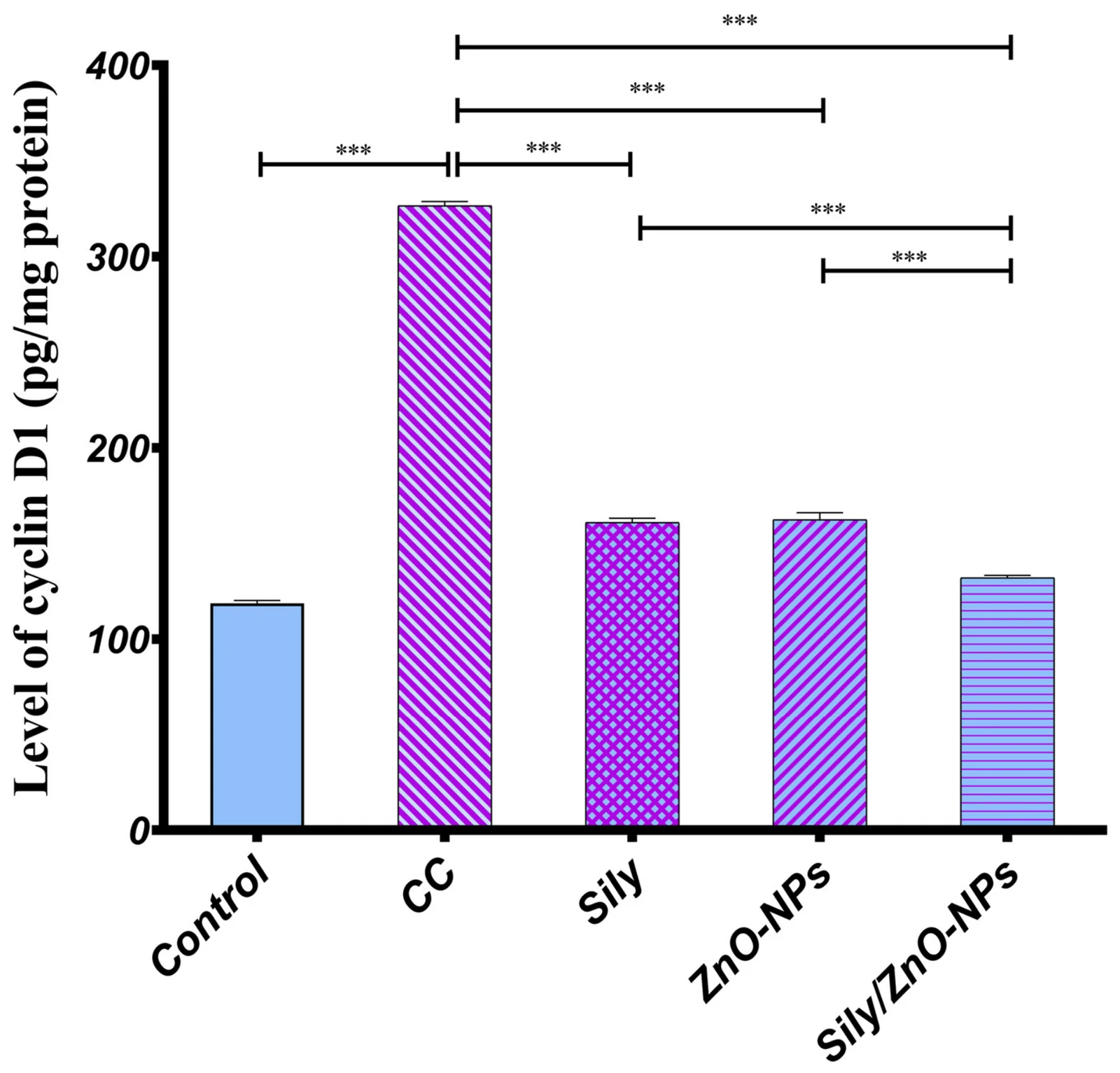
Sily and ZnO-NPs exert antiproliferative activity against DMH-induced CC. The antiproliferative activity of Sily, ZnO-NPs, and Sily/ZnO-NPs was assessed by measuring the level of cyclin D1 (pg/mg protein) by ELISA in different groups. Data are presented as the mean ± standard error of the mean (SEM). *** *p* ≤ 0.001.

To explore the antiproliferative activity of Sily and ZnO-NPs on DMH-induced CC, the cyclin-D1 protein level was assessed. The protein level of cyclin-D1 was expressively increased by 174.9% (*p* ≤ 0.001) in the DMH-induced CC group compared with the control group. Remarkably, Sily, ZnO-NPs, and Sily/ZnO-NPs reduced the level of cyclin-D1 by 50.7%, 50%, and 59.6% (*p* ≤ 0.001), respectively, with respect to the DMH-induced CC group. Also, Sily/ZnO-NPs reduced the level of cyclin-D1 by 17.9% and 18.7% (*p* ≤ 0.001), respectively, compared with Sily and ZnO-NPs, respectively (Figure 8).

**Figure 9:**
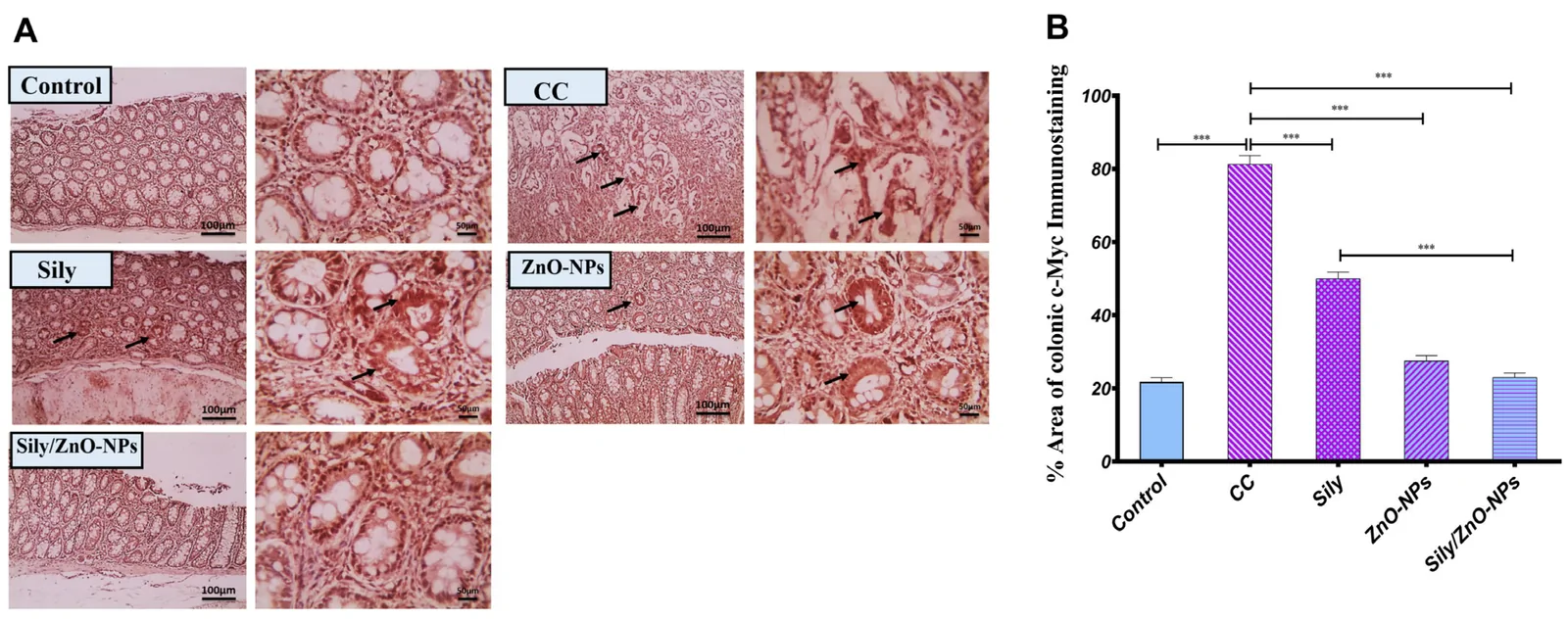
Effects of Sily and ZnO-NPs on c-myc protein expression in tumor tissue determined by immunohistochemical (IHC) staining. (**A**) Representative images of anti-c-myc-stained colon sections. Black arrows point to positive expressions. IHC counterstained with Mayer’s hematoxylin. Magnifications X: 100 bar 100 and X: 400 bar 50. (**B**) Quantitative analysis of c-myc expression represented as percentage area (%). Data are presented as the mean ± standard error of the mean (SEM). *** *p* ≤ 0.001.

Microscopic pictures of immunostained colonic sections showed markedly increase in the c-Myc expression in the crypt epithelium in the CC group by 273.6% (*p* ≤ 0.001) with respect to the control group. Colonic sections from Sily-, ZnO-NP-, and Sily/ZnO-NP-treated groups showed significant reductions in the c-Myc expression in the crypt epithelium by 38.5%, 66.2%, and 71.7% (*p* ≤ 0.001), with respect to the CC group, respectively. Colonic sections from the Sily/ZnO-NP combination-treated group exhibited a marked reduction in the expression of c-Myc in the crypt epithelium by 54% (*p* ≤ 0.001) with respect to the Sily group (Figure 9).

### 3.7. Sily and ZnO-NPs Mitigate DMH-Induced Oxidative Stress and Exert Antioxidant Activity

The rats with induced CC showed significant changes in the oxidative status of colon tissues, with a significant reduction in antioxidant defense system parameters (GSH and SOD) by 72.3% and 42.8% (*p* ≤ 0.001), respectively, and an elevation in oxidative stress (MDA) by 395% (*p* ≤ 0.001) as compared with the control group. Fortunately, treatment of CC-modeled rats with Sily and Sily/ZnO-NPs led to a significant compensation of the diminished GSH content by 97% (*p* < 0.05) and 156.8% (*p* ≤ 0.01), respectively, and upregulated the activity of colon SOD by 48.6% (*p* ≤ 0.01) and 57.8% (*p* ≤ 0.001), respectively, as compared with the CC group.

**Figure 10:**
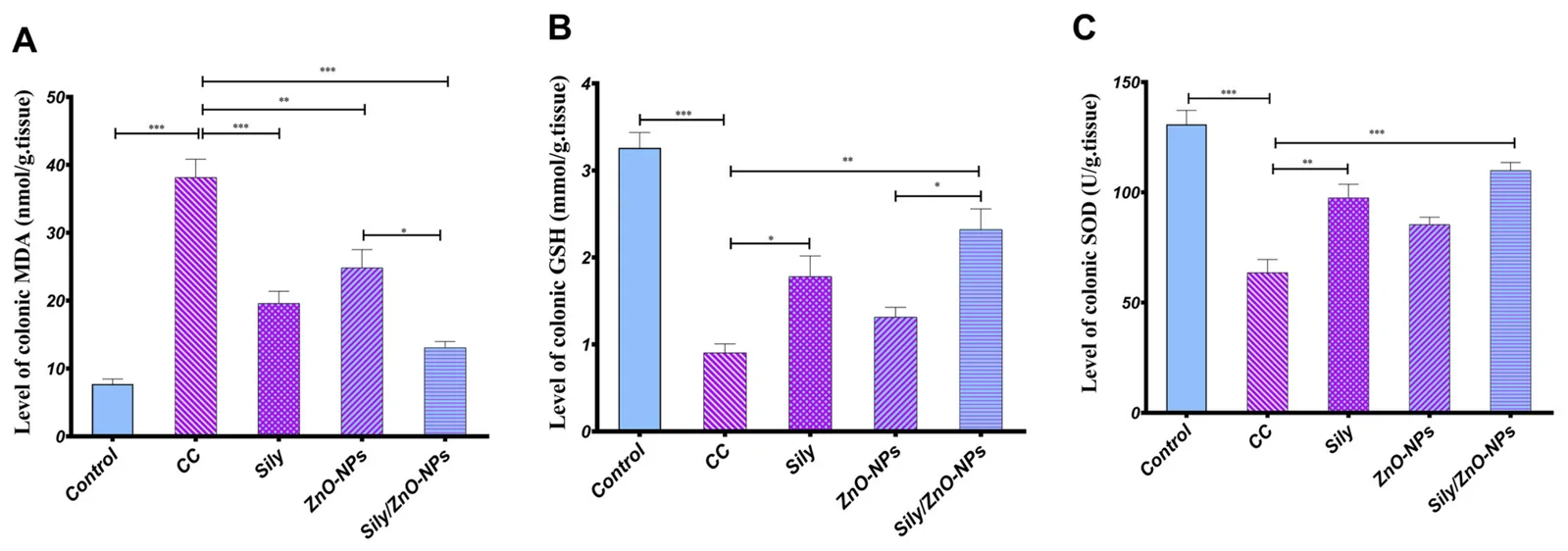
Sily and ZnO-NPs mitigate DMH-induced oxidative stress and exert antioxidant activity. Oxidative stress in colon tissues was evaluated by determining thr levels of MDA (**A**), GSH (**B**), and SOD (**C**). Data are presented as the mean ± standard error of the mean (SEM). Statistical significance is indicated as follows: * *p* < 0.05, ** *p* ≤ 0.01, and *** *p* ≤ 0.001.

Promisingly, Sily, ZnO-NPs, and Sily/ZnO-NPs succeeded in the downregulation of colon MDA levels by 48.6% (*p* ≤ 0.001), 34.8% (*p* ≤ 0.01), and 65.7% (*p* ≤ 0.001) compared to the corresponding values of the CC group. Also, Sily/ZnO-NPs caused a significant elevation in the GSH level by 76.6% (*p* < 0.05) and a significant reduction in the MDA level by 47.4% (*p* < 0.05), as compared with the ZnO-NP group (Figure 10).

### 3.8. Sily and ZnO-NPs Mitigate DMH-Induced Invasion and Metastasis

**Figure 11:**
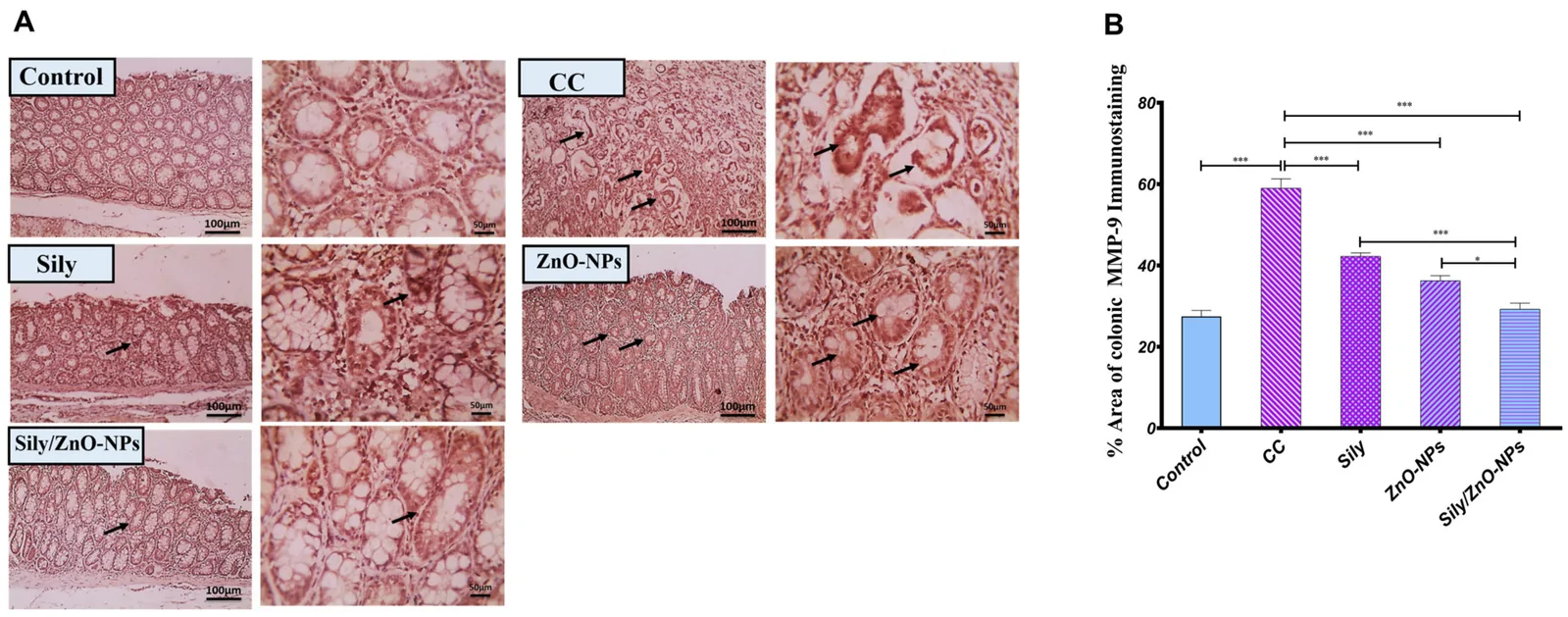
Sily and ZnO-NPs mitigate DMH-induced invasion and metastasis. The effect of Sily and ZnO-NPs against DMH-induced invasion and metastasis was assessed by measuring MMP-9 protein expression in different groups by immunohistochemical (IHC) staining. (**A**) Representative images of anti-MMP-9-stained colon sections. Black arrows point to positive expressions. IHC counterstained with Mayer’s hematoxylin. Magnifications X: 100 bar 100 and X: 400 bar 50. (**B**) Quantitative analysis of MMP-9 expression represented as the percentage area (%). Data are presented as the mean ± standard error of the mean (SEM). * *p* < 0.05; *** *p* ≤ 0.001.

Microscopic pictures of immunostained colonic sections showed markedly increased expression of MMP-9 in the crypt epithelium in the DMH-induced CC group by 114.5% (*p* ≤ 0.001) with respect to the control group. Colonic sections from Sily-, ZnO-NP-, and Sily/ZnO-NP-treated groups resulted in significantly lower MMP-9 expression in the crypt epithelium by 28.4%, 38.6%, and 50.4% (*p* ≤ 0.001) with respect to the CC group, respectively. Colonic sections from the Sily/ZnO-NP combination-treated group showed a marked decline in MMP-9 expression by 30.8% (*p* ≤ 0.001) and 19.3% (*p* < 0.05), in comparison with the Sily and ZnO-NP groups, respectively (Figure 11).

### 3.9. Sily and ZnO-NPs Mitigate DMH-Induced Colon Stemness

**Figure 12:**
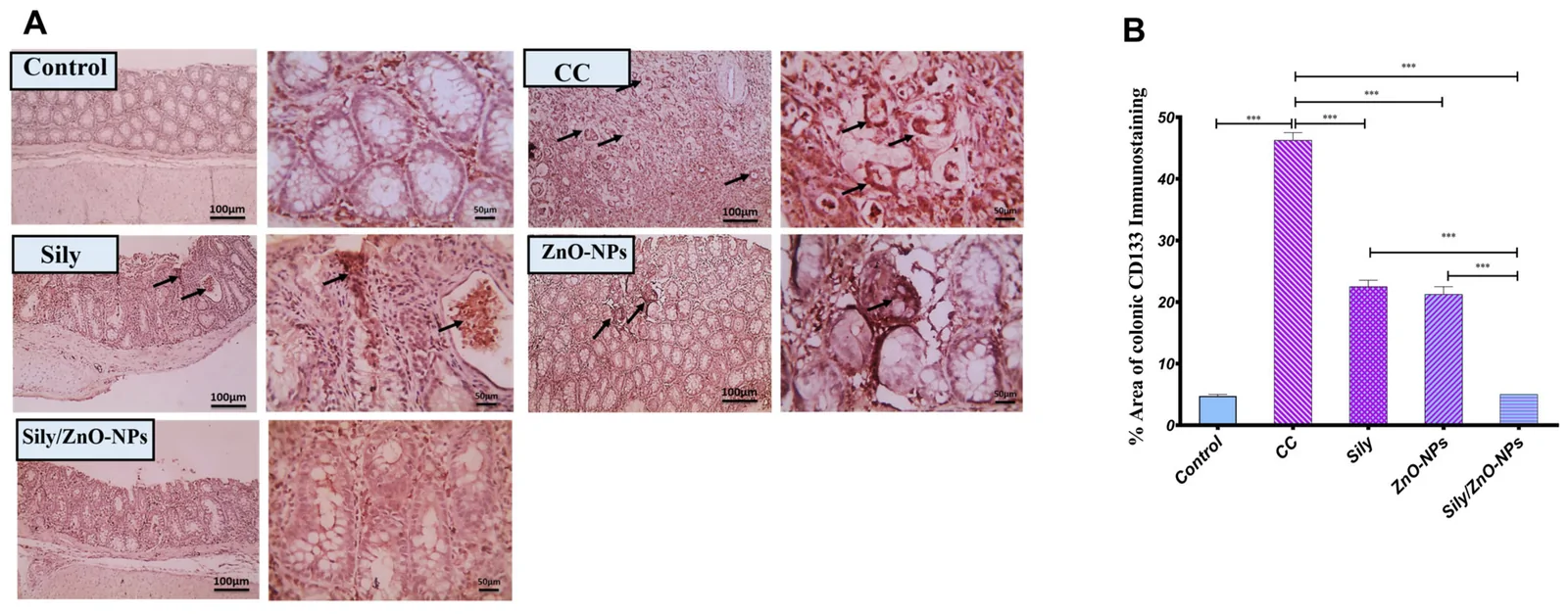
Sily and ZnO-NPs mitigate DMH-induced colon stemness. The effect of Sily and ZnO-NPs against DMH-induced colon stemness was assessed by measuring CD133 protein expression in different groups by immunohistochemical (IHC) staining. (**A**) Representative images of anti-CD133-stained colon sections. Black arrows point to positive expressions. IHC counterstained with Mayer’s hematoxylin. Magnifications X: 100 bar 100 and X: 400 bar 50. (**B**) Quantitative analysis of CD133 expression represented as the percentage area (%). Data are presented as the mean ± standard error of the mean (SEM). *** *p* ≤ 0.001.

Microscopic pictures of immunostained colonic sections showed negative expression of CD133 in the crypt epithelium in the control group. In contrast, colonic sections from CC group demonstrated a significant elevation in CD133 expression in the crypt epithelium by 873.7% (*p* ≤ 0.001) with respect to the control group. Colonic sections from Sily-, ZnO-NP-, and Sily/ZnO-NP-treated groups showed a marked decline in the expression of CD133 in the crypt epithelium by 51.4%, 54%, and 89.2% (*p* ≤ 0.001) with respect to the CC group, respectively. Colonic sections from the Sily/ZnO-NP combination-treated group demonstrated a significant decline in the expression of CD133 by 77.8% and 76.5% (*p* ≤ 0.001), with respect to the Sily and ZnO-NP groups, respectively (Figure 12).

### 3.10. Multivariable Data Analyses

**Figure 13:**
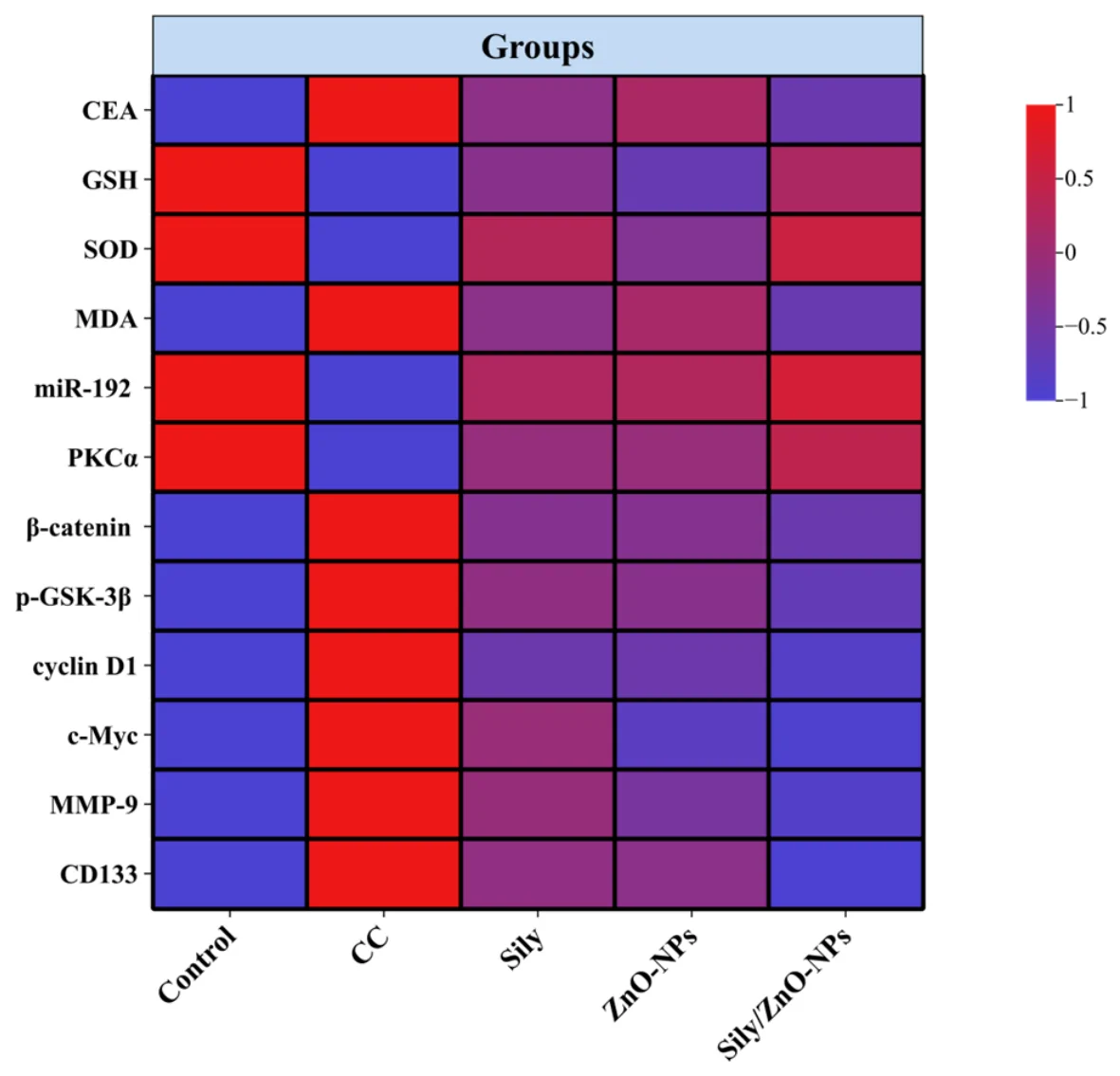
Heatmap illustrating biochemical, oxidative stress, and molecular markers across the control, CC, Sily, ZnO-NP, and Sily/ZnO-NP groups. Color gradients represent relative expression levels (red = upregulation, orange = downregulation; Z-score normalized). The CC group showed marked elevations in CEA, MDA, β-catenin, p-GSK-3β, cyclin D1, c-Myc, MMP-9, and CD133. In contrast, these markers were substantially reduced in the control, Sily, ZnO-NP, and Sily/ZnO-NP groups. Conversely, GSH, miR-192, and PKCα were upregulated in the Sily, ZnO-NP, and Sily/ZnO-NP groups, indicating improved redox status and therapeutic effects against CC progression.

Hierarchical clustering combined with heatmap visualization of the assessed biochemical and molecular markers demonstrated distinct patterns across the experimental groups (Control, CC, Sily, ZnO-NPs, and Sily/ZnO-NPs). The dark red coloration demonstrates that the CC group displayed elevated levels of tumor and oxidative stress markers, including CEA, MDA, β-catenin, p-GSK-3β, cyclin D1, c-Myc, MMP-9, and CD133. In contrast, the Control group showed markedly lower levels of these markers (orange gradient), reflecting reduced tumor progression and oxidative stress. Conversely, antioxidant and regulatory molecules, such as SOD, GSH, miR-192, and PKCα, displayed elevated expression in the control group. The observed clustering patterns reveal the modulatory effects of Sily, ZnO-NPs, and their combination (Sily/ZnO-NPs) on tumor biomarkers and oxidative stress (Figure 13).

### 3.11. STRING Reveals Crosstalk Among PKCα, β-Catenin, and Stemness-Associated Proteins

**Figure 14:**
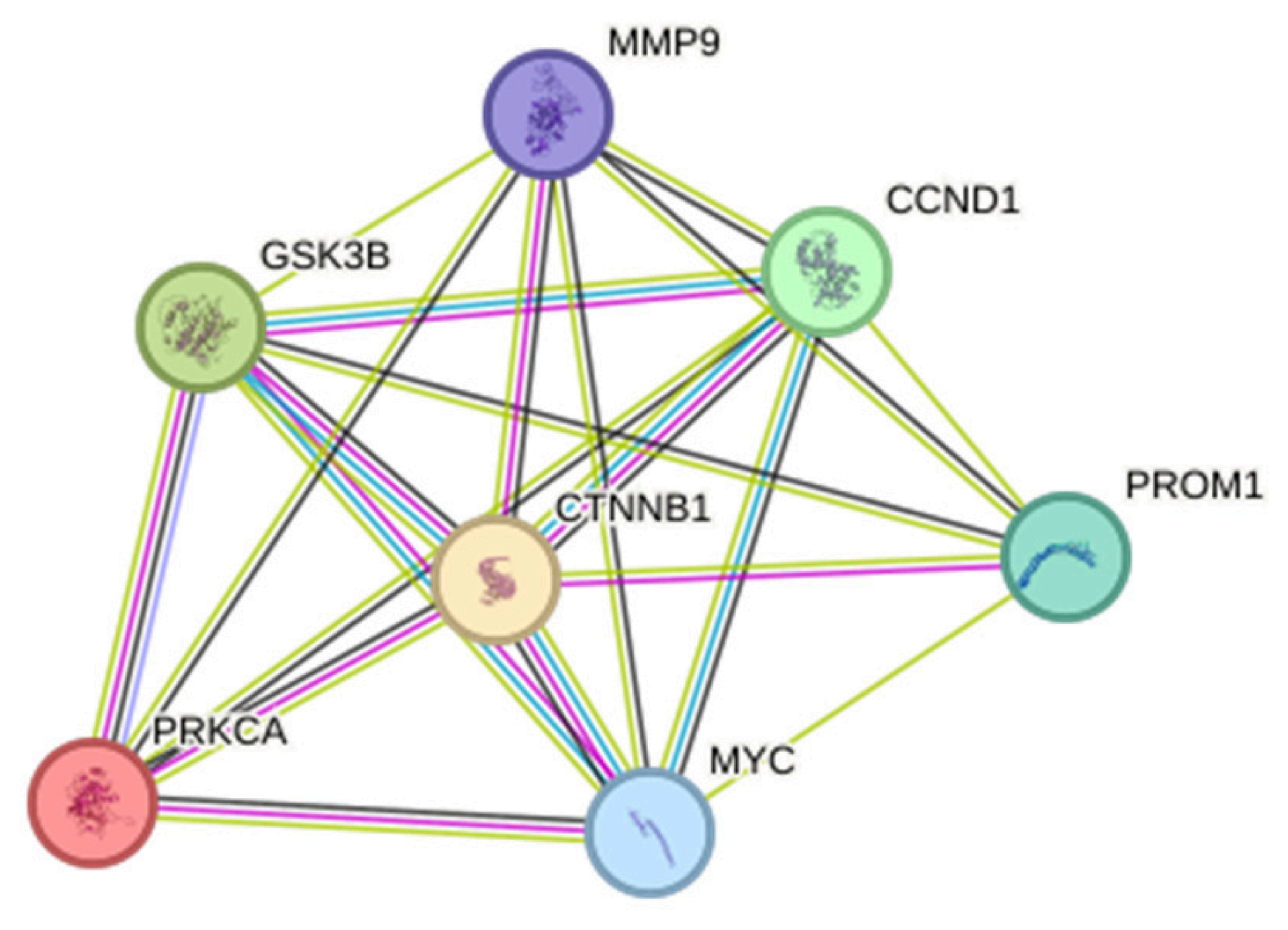
STRING protein–protein interaction (PPI) network.

The STRING analysis revealed a highly interconnected network among PKCα, β-catenin, GSK-3β, cyclin D1, c-Myc, MMP-9, and CD133. Strong functional associations were evident, particularly the β-catenin/GSK-3β/cyclin D1/c-Myc axis, indicating coordinated regulation of cell proliferation pathways. PKCα showed direct links with β-catenin and MMP-9, supporting its role in invasion signaling. CD133 connected indirectly through β-catenin-related pathways, suggesting involvement in stemness-associated signaling. The dense pattern of colored evidence lines confirmed high-confidence protein–protein interactions in this regulatory network (Figure 14).

### 3.12. miR-192 Upregulation Induced by Sily, ZnO-NPs, and Sily/ZnO-NPs Correlates with β-Catenin and p-GSK-3β Downregulation and PKCα Upregulation

**Figure 15:**
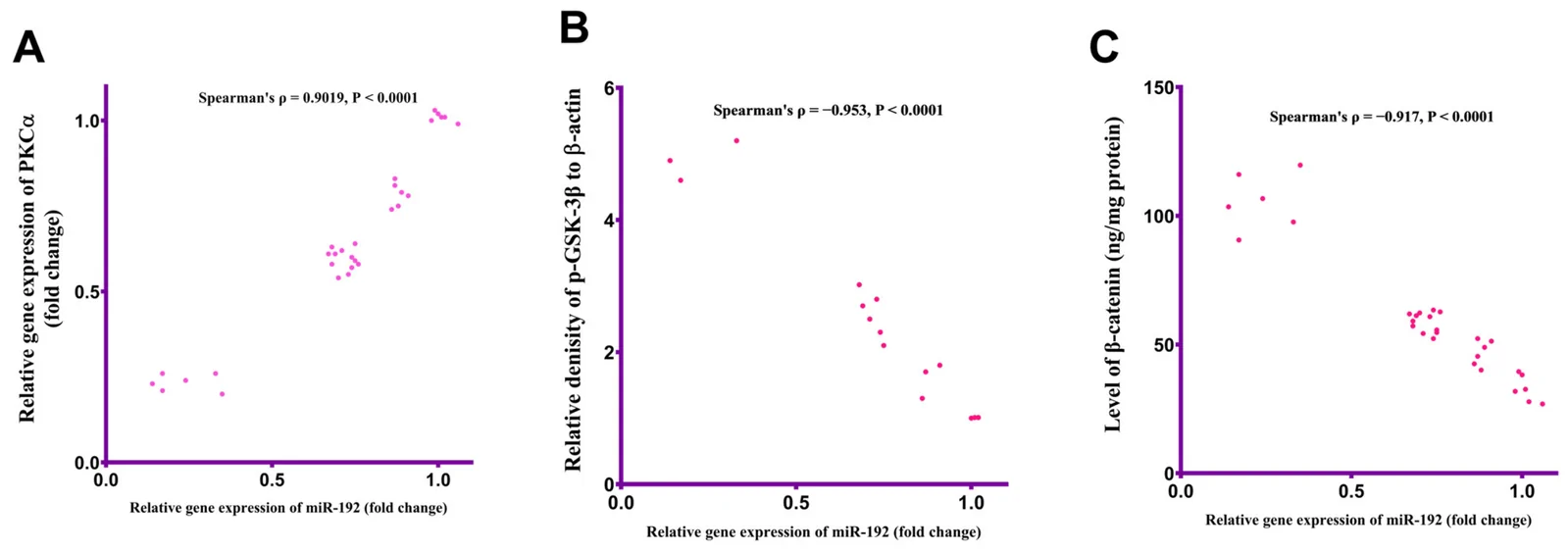
miR-192 upregulation induced by Sily, ZnO-NPs, and Sily/ZnO-NPs correlates with β-catenin and p-GSK-3β downregulation and PKCα upregulation. (**A**) miR-192 expression shows a positive correlation with PKCα gene expression. (**B**) miR-192 expression is inversely correlated with p-GSK-3β protein levels. (**C**) miR-192 expression is inversely associated with β-catenin protein levels.

Statistical analysis revealed a strong positive correlation between miR-192 and PKCα (ρ = 0.902, *p* < 0.0001) (Figure 15A), whereas miR-192 exhibited significant negative correlations with either p-GSK-3β (ρ = −0.9526, *p* < 0.0001) or β-catenin (ρ = −0.917, *p* < 0.0001) (Figure 15B,C).

## 4. Discussion

Colon cancer continues to be a significant worldwide health concern because of its high prevalence and quick progression and the limited effectiveness of current treatment strategies [50,51]. Chemical carcinogens such as DMH stimulate and promote colon tumor formation through oxidative stress, DNA damage, chronic inflammation, and disruption of key signaling pathways [52,53]. These changes collectively lead to uncontrolled cell proliferation, tissue invasion, and activation of cancer stem cell (CSC) populations, which are hallmarks that complicate treatment and worsen the prognosis [54]. Given the multifactorial nature of colon cancer, therapeutic techniques are desperately needed capable of targeting multiple mechanistic pathways simultaneously.

In this context, natural antioxidants and nanomaterial-based interventions have emerged as promising options. Sily, a plant-derived flavonoid compound, exhibits potent antioxidant, anti-inflammatory, and antiproliferative properties [55]; meanwhile, ZnO-NPs work to enhance the cells’ antioxidant defenses and modify molecular pathways associated with tumor suppression [56]. Combining these two agents provides a novel therapeutic strategy designed to offer complementary protection by alleviating oxidative stress, inhibiting proliferation and invasion, and targeting CSC signaling. Accordingly, assessing how Sily, ZnO-NPs, and their combination modulate the biochemical and molecular changes induced by DMH is essential to understanding their chemopreventive potential.

The marked increase in CEA levels was a rapid indicator of what was being invented in this model and a clinically significant indicator of the increasing diversity in colorectal cancer (CRC) [57]. Chronic DMH administration (20 mg/kg/week for 16 weeks) resulted in a marked elevation in serum CEA levels compared to the control group, reflecting increased proliferative activity, a loss of epithelial cell differentiation, and the release of tumor-associated glycoprotein [37]. The therapeutic intervention using Sily, ZnO-NPs, and their combination significantly reduced this elevation. The enhanced effect of the combined treatment is likely due to a complementary interaction between Sily’s bioactive flavonolignans [58] and the unique physical and chemical properties of ZnO-NPs [59,60], which together contribute to an enhanced antioxidant capacity, attenuation of the signaling pathways associated with malignant transformation, and the reduction of tumor antigen release.

Evaluation of oxidative stress biomarkers in colon tissue demonstrated marked alterations in redox homeostasis following DMH-induced colon cancer. CC rats showed a significant depletion of endogenous antioxidant defenses, characterized by marked reductions in GSH levels and SOD activity alongside a notable elevation in lipid peroxides (MDA) compared to control group, suggesting oxidative damage that may promote tumor development [61]. DMH is metabolically activated in the liver to form reactive mediators that generate ROS in excess, leading to damage to DNA, lipids, and proteins [62]. This oxidative damage can lead to mutations, disrupt cellular signaling, and create a microenvironment conducive to tumor growth [63], which is consistent with the marked increase in CEA levels, reflecting an increased tumor burden and malignant transformation.

Treatment with Sily or a combination of Sily/ZnO-NPs effectively restored the redox balance, resulting in a marked increase in GSH levels and SOD activity, with the combination therapy demonstrating superior efficacy. Sily exerts its antioxidant effect by eliminating ROS, upregulating endogenous antioxidant enzymes, and stabilizing cell membranes [64]. In contrast, ZnO-NPs alone significantly reduced MDA levels by attenuating lipid peroxidation, and they did not significantly restore depleted GSH content or SOD activity on their own. It is important to note that while ZnO-NPs frequently manifest pro-oxidant, tumor-selective cytotoxicity through localized ROS generation, the reductions in MDA observed here in whole-colon tissue homogenates reflect an overall improvement in tissue redox homeostasis and a decrease in lipid peroxidation-associated tissue damage, rather than a uniform suppression of ROS across all cell populations. These apparently contrasting effects, localized tumor cytotoxicity versus whole-tissue protection, are not mutually exclusive and reflect the context-, dose-, and cell-type-dependent redox behavior of ZnO-NPs, supported by their reported radical-scavenging capabilities and ability to modulate antioxidant defenses under specific conditions [65,66]. All treatments reduced MDA levels, with the combination therapy having the greatest effect, demonstrating complementary protection of colon tissue from DMH-induced oxidative damage while maintaining its selective toxicity against tumorigenic cells. Thus, the enhanced antioxidant profile of the combination therapy likely stems from the combined, complementary actions of Sily in restoring endogenous antioxidant enzyme defenses and ZnO-NPs in suppressing oxidative lipid peroxidation at the tissue level while acknowledging that whole-tissue biochemical measurements inherently lack the resolution to capture cell-type-specific or transient ROS dynamics within tumor compartments.

Histopathological findings further confirmed the biochemical findings and provided direct morphological evidence of DMH-induced colon carcinogenesis and the protective effects of the therapies. Control colon sections showed a normal architecture, with intact mucosal, submucosal, and muscular layers and well-organized epithelial cells rich in mucin-secreting goblet cells. In contrast, rats treated with DMH showed pronounced tumor changes, including invasive adenocarcinoma extending into the muscularis mucosae, high-grade dysplastic crypts, marked edema, and dense infiltration of inflammatory cells, reflecting active inflammation and aggressive tumor progression.

Therapeutic interventions using Sily, ZnO-NPs, and their combined formulation have significantly alleviated these pathological changes, prevented DMH-induced cellular transformation, and preserved the structural integrity of the colon. Rats treated with Sily showed moderate dysplasia and marked infiltration of inflammatory cells, indicating partial protection but with residual neoplastic changes. This is consistent with previous reports showing that Sily alleviates colonic dysplasia and stabilizes epithelial structure through its antioxidant and anti-inflammatory properties [67]. Treatment with ZnO-NPs resulted in low-grade dysplasia, which is consistent with some studies suggesting that ZnO-NPs provide structural protection by enhancing antioxidant defenses [39]. It is noteworthy that the combination of Sily/ZnO-NPs showed an almost complete restoration of the normal architecture of the colon while preserving mucosal, submucosal, and muscular layers that closely resemble control tissue.

PAS staining results provided additional insight into mucosal integrity and goblet cell function across experimental groups. Control colon sections showed a well-preserved glandular architecture with an abundance of PAS-positive goblet cells, indicating healthy mucin production and intact mucosal defense. In contrast, rats treated with DMH showed a marked decrease in PAS-positive mucin in the crypt epithelium, reflecting impaired goblet cell function and a compromised epithelial barrier. Mechanistically, DMH disrupts goblet cell balance by stimulating oxidative and inflammatory signaling that reduces mucin gene expression, alters secretory differentiation, and accelerates epithelial cell renewal [62,68]. These mechanistic changes are consistent with our biochemical findings showing elevated MDA levels and decreased GSH content and SOD activity in DMH-treated rats, supporting the view that DMH-induced oxidative stress is a major driver of mucosal injury [69]. Therefore, decreased mucin production increases susceptibility to inflammation and tumor development [70]. Sily and ZnO-NP treatments resulted in the restoration of mucin secretion. The combined treatment resulted in near-complete restoration of PAS-positive mucin, likely achieved through the complementary actions of Sily in supporting endogenous antioxidant defenses and ZnO-NPs in attenuating lipid peroxidation, thereby enhancing mucosal barrier function [71,72].

miR-192 has been found to act as a tumor suppressor in a variety of cancers, including CC [18,73,74]. As one of the p53-induced miRNAs [75,76], miR-192 is involved in cancer development and progression by targeting SRPX2 [77] and RhoA [73]. Consistent with this, the CC group showed a significant decrease in miR-192 expression. Similarly, substantial evidence indicates that PKCα acts as a tumor suppressor in intestinal epithelial cells. PKCα expression is frequently decreased in human CRC, and its downregulation induces intestinal tumorigenesis in mouse models [78,79], whereas the activation of PKCα significantly reduces CRC cell invasion and growth [80]. In agreement with these findings, PKCα levels were significantly decreased in the CC group.

The simultaneous reduction of miR-192 and PKCα in colon tissue after DMH exposure reflects carcinogen-induced oxidative DNA damage, disruption of epithelial differentiation, and altered microRNA processing. These concurrent molecular alterations mirror the tumor-suppressive roles that miR-192 plays in cell-cycle regulation, DNA damage response, and epithelial integrity [81] alongside the essential role of PKCα in maintaining controlled proliferation and intestinal tissue homeostasis under physiological conditions [82], which are cooperatively disrupted under oxidative stress induced by DMH, establishing a correlative framework for understanding early carcinogenic shifts rather than a direct regulatory mechanism.

Treatment with Sily significantly increased expression of both miR-192 and PKCα compared with the carcinogenic control. This observation agrees with previous studies demonstrating that Sily can modulate tumor-suppressive microRNAs, restore epithelial signaling, and counteract carcinogen-induced oxidative damage [83]. Sily has also been reported to regulate miRNA expression, including the downregulation of oncogenic miRNAs such as miR-21, thereby influencing cell-cycle regulation and reversing epigenetic dysregulation [84]. Additionally, Silibinin has been shown to increase PKCα protein levels and enhance kinase-mediated differentiation in cancer cells [85].

Similarly, rats treated with ZnO-NPs exhibited a pronounced increase in miR-192 and PKCα expression, exceeding that of Sily alone. These results are in agreement with previous studies demonstrating that Zn supplementation or NP formulations improve redox balance, enhance DNA repair, and regulate transcription factors responsible for microRNA synthesis [86,87]. Zn is also known to participate in microRNA maturation machinery and preserve PKC-dependent signaling pathways, especially under conditions of oxidative challenge [88,89]. The superior effect of ZnO-NPs compared with Sily alone may be attributed to their high bioavailability, improved cellular uptake, and the essential role of Zn as a cofactor in transcriptional and enzymatic processes regulating colonic mucosal homeostasis [39,90].

Notably, the combined Sily/ZnO-NP treatment produced the most substantial elevation in both miR-192 and PKCα levels, indicating a clear complementary interaction between the two agents. This enhanced response is consistent with studies reporting that combining natural antioxidants with nanoformulated minerals yields amplified effects on gene expression, redox regulation, and molecular repair mechanisms [91,92].

Previous studies reported that miR-192 regulates the Wnt/β-catenin pathway [93], and GSK3 is a key signaling molecule in cancer as well as Wnt/β-catenin and regulates c-Myc [94]. Moreover, PKCα can exert its tumor suppressive activity in the intestinal epithelium by antagonizing the activity of the Wnt/β-catenin signaling pathway, with its activity gradually increasing from the crypt to the surface in the normal intestinal epithelium but significantly reducing in CRC, in which the Wnt/β-catenin signaling pathway is constitutively activated [78,95]. PKCα inhibits Wnt/β-catenin pathway through two mechanisms: phosphorylation of the orphan receptor RORα, which represses β-catenin co-transcriptional activity [96], and direct phosphorylation of β-catenin, promoting its ubiquitination and degradation by the proteasome [97].

Based on the aforementioned data, the evaluation of β-catenin and p-GSK-3β, together with supportive molecular docking findings, provides preliminary insights into possible interactions through which miR-192 and PKCα may exert their inhibitory effects on colon carcinogenesis. In the present study, DMH administration led to a substantial upregulation of β-catenin and p-GSK-3β compared with the control group. This pronounced activation of the Wnt/β-catenin pathway is a hallmark of DMH-induced tumorigenesis, as excessive β-catenin accumulation promotes uncontrolled proliferation, resistance to apoptosis, and enhanced neoplastic transformation [98,99]. The concurrent elevation of p-GSK-3β further contributes to carcinogenic progression, as phosphorylation inactivates GSK-3β, thereby preventing β-catenin degradation and allowing its nuclear translocation to activate oncogenic transcription [100,101].

Treatment with Sily, ZnO-NPs, and particularly their combination markedly reversed the DMH-induced activation of the Wnt/β-catenin pathway. All treatments resulted in a marked decrease in β-catenin levels and a decrease in p-GSK-3β levels, indicating the robust restoration of GSK-3β activity and suppression of aberrant proliferation signals. These biochemical improvements are supported by the molecular docking findings, which predicted strong binding interactions between Sily and GSK-3β (binding affinity –9.5 kcal/mol) through stabilizing hydrogen bonds and hydrophobic interactions, while ZnO-NPs also showed a possible interaction with GSK-3β but with lower affinity. These in silico predictions offer a plausible framework for the observed effects, acknowledging that experimental target-engagement studies are required for ultimate validation. As confirmation of these findings, Eo et al., (2016) reported that Sily activated proteasomal degradation to downregulate β-catenin protein in CRC cells [102]. Awadalla et al., (2022) observed that ZnO-NPs caused downregulation of β-catenin proteins in adenine-fed rats [103]. Meanwhile, LiCl (a β-catenin activator) could significantly reverse the ZnO-NP-induced osteosarcoma cell proliferation, migration, invasion, and EMT inhibition [104]. The superior reductions observed with the combined Sily/ZnO-NP therapy highlight a complementary mechanism consistent with Sily’s strong kinase-binding potential and Zn’s regulatory role in kinase activation. Taken together, these results underscore the strong cooperative ability of Sily and ZnO-NPs to restore GSK-3β function, enhance β-catenin degradation, and counteract DMH-induced cancer signaling.

The enhanced suppression of Wnt/β-catenin signaling in the combination group coincides with the upstream restoration of miR-192 and PKCα, both of which play key regulatory roles in epithelial homeostasis and carcinogenesis [81,105]. miR-192 is a known inhibitor of Wnt pathway activation [93], and PKCα can enhance β-catenin degradation through phosphorylation-dependent mechanisms [106]. Thus, the observed increases in miR-192 and PKCα following treatment with Sily and ZnO-NPs may be attributed to the downstream inhibition of β-catenin and reactivation of GSK-3β. However, functional validation remains necessary to establish direct causality.

The correlation analysis further strengthens this associative framework. A strong positive correlation was observed between miR-192 and PKCα, indicating that both molecules respond in a coordinated manner as part of a related expression pattern. This relationship is consistent with previous evidence indicating that miR-192 co-occurs with signaling pathways that relate to PKCα expression, contributing to epithelial cell homeostasis and tumor suppression [18]. Conversely, miR-192 demonstrated strong negative correlations with both p-GSK-3β and β-catenin, indicating that higher miR-192 expression is closely associated with reduced activation of the Wnt/β-catenin signaling cascade. Such an association aligns with previous studies showing that miRNAs, including miR-192, are closely linked to the suppression of colon tumor progression by modulating the Wnt/β-catenin signaling pathway and regulating β-catenin stability and transcriptional activity [107,108]. The strength of these correlations highlights the potential involvement of miR-192 in regulating this carcinogenic pathway and underscores that establishing a direct regulatory hierarchy requires dedicated functional rescue studies.

The antiproliferative effects of Sily, ZnO-NPs, and their combination were further substantiated by assessing cyclin D1 expression, a central regulator of the G1/S transition in the cell cycle [109]. DMH-induced CC caused a marked upregulation of cyclin D1 protein levels relative to the control group, reflecting an enhanced proliferative drive and dysregulated cell-cycle progression characteristic of neoplastic transformation [110]. Treatment with Sily or ZnO-NPs significantly counteracted this effect, demonstrating their capacity to suppress DMH-induced cell-cycle disturbances. Importantly, the combined Sily/ZnO-NPs therapy produced the strongest response, yielding a greater reduction in cyclin D1 than either treatment alone. This complementary effect aligns with the observed downregulation of β-catenin and upregulation of miR-192/PKCα, suggesting that the enhanced antiproliferative activity of the combination therapy is associated with the concurrent modulation of Wnt/β-catenin signaling and its associated cell-cycle regulatory pathways.

Parallel to these findings, immunohistochemical analysis revealed a marked elevation in c-Myc expression in the DMH group compared with controls. This pronounced overexpression, particularly within the crypt epithelium, indicates intensified mitogenic signaling and accelerated cell-cycle progression characteristic of DMH-induced tumorigenesis [111]. As a well-established downstream target of the Wnt/β-catenin pathway, c-Myc plays a central role in driving uncontrolled cell proliferation, metabolic reprogramming, and impaired differentiation [112]. Accordingly, the widespread immunoreactivity of c-Myc reflects the ongoing activation of cancer signaling cascades in the colonic crypts, the main proliferative compartment of the intestinal epithelium [113].

Treatment interventions mitigated this response to varying degrees. Sily modestly reduced c-Myc expression, whereas ZnO-NPs produced a more pronounced inhibitory effect. Notably, the Sily/ZnO-NPs combination achieved the most substantial reduction, restoring expression to near-normal levels. Mechanistically, Sily exerts its antiproliferative effects in association with the modulation of the Wnt/β-catenin pathway and the activation of GSK-3β, thereby promoting cyclin D1 degradation and suppressing proliferative signaling, consistent with the findings of our study and previous reports [114,115]. Likewise, ZnO-NPs have been reported to exert antiproliferative effects via oxidative stress-mediated signaling and interference with tumorigenic cell-cycle regulators [116]. The superior efficacy of the combined Sily/ZnO-NP formulation likely reflects complementary interactions that coincide with enhanced β-catenin inhibition and stronger co-modulation of cyclin D1 and c-Myc. However, dedicated functional validation remains necessary to confirm direct target engagement. Overall, these findings indicate that combined Sily and ZnO-NP therapy more effectively suppresses c-Myc in parallel with Wnt/β-catenin-driven cell-cycle dysregulation, thereby offering superior protection against DMH-induced colon tumor growth compared with monotherapy.

Immunohistochemical analysis revealed a marked increase in MMP-9 expression in the crypt epithelium of DMH-treated rats compared to the controls, indicating increased extracellular matrix degradation and enhanced invasive capacity [117]. Sily treatment resulted in a moderate decrease in MMP-9 expression, while ZnO-NPs exerted a more pronounced inhibitory effect. It is noteworthy that the combined treatment with Sily/ZnO-NPs significantly reduced MMP-9 immunopositivity to near-normal levels, highlighting the complementary efficacy of the two agents in suppressing matrix remodeling and mitigating markers associated with CC development.

The immunohistochemical analysis of colonic sections revealed a marked alteration in CD133 expression, a well-established CSC marker [118], across experimental groups. In control tissues, CD133 was virtually absent in the crypt epithelium, consistent with the low prevalence of stem-like tumorigenic cells in healthy colon tissue. In contrast, the DMH group exhibited a dramatic upregulation of CD133, indicating a substantial enrichment of CSCs, which are known to drive tumor initiation, progression, and chemoresistance [119]. Treatment with Sily led to a marked reduction of CD133 expression in both the crypt epithelium and lumen, indicating partial suppression of CSC populations. ZnO-NPs similarly decreased CD133 levels in the crypt epithelium, reflecting effective targeting of stem-like tumor cells. Notably, the combined Sily/ZnO-NP therapy almost completely abolished CD133 expression, restoring it to a pattern comparable to normal control tissue. These findings suggest that both agents can target CSCs by inhibiting self-renewal signaling, promoting differentiation, or inducing apoptosis [120,121,122,123]. The combination therapy exhibited superior efficacy over either monotherapy, demonstrating a complementary effect in depleting the CSC population.

The hierarchical clustering and heatmap analysis clearly differentiated the experimental groups, with the CC group exhibiting a distinct profile characterized by strong upregulation of tumorigenic and oxidative stress markers (CEA, MDA, β-catenin, p-GSK-3β, cyclin D1, c-Myc, MMP-9, and CD133). This pattern confirms the biochemical findings showing severe oxidative imbalance and activation of the Wnt/β-catenin proliferative axis in DMH-induced CC. In contrast, the control group showed low expression of these markers alongside preserved antioxidant capacity and regulatory mechanisms (manifested by maintained GSH content, SOD activity, and miR-192 and PKCα expression), reflecting normal cellular homeostasis.

The treatment groups shifted toward the control cluster, with Sily, ZnO-NPs, and particularly the Sily/ZnO-NP combination reducing tumor markers and restoring antioxidant defenses. The Sily/ZnO-NP group displayed the closest resemblance to the control profile, in line with earlier results showing a superior ability to downregulate β-catenin, cyclin D1, and c-Myc, suppress MMP-9 and CD133, as well as restore GSH content, SOD activity, and miR-192 expression. These clustering patterns support the STRING network analysis, which identified a tightly interconnected signaling axis involving β-catenin, GSK-3β, cyclin D1, and c-Myc and highlighted PKCα as a key regulator linked to β-catenin and MMP-9. Together, the heatmap, biochemical, and network analyses suggest a mechanism by which the Sily/ZnO-NP combination helps to restore tissue redox homeostasis, modulates proliferative signaling, and targets the interconnected molecular network driving DMH-induced colon carcinogenesis.

## 5. Conclusions

**Figure 16:**
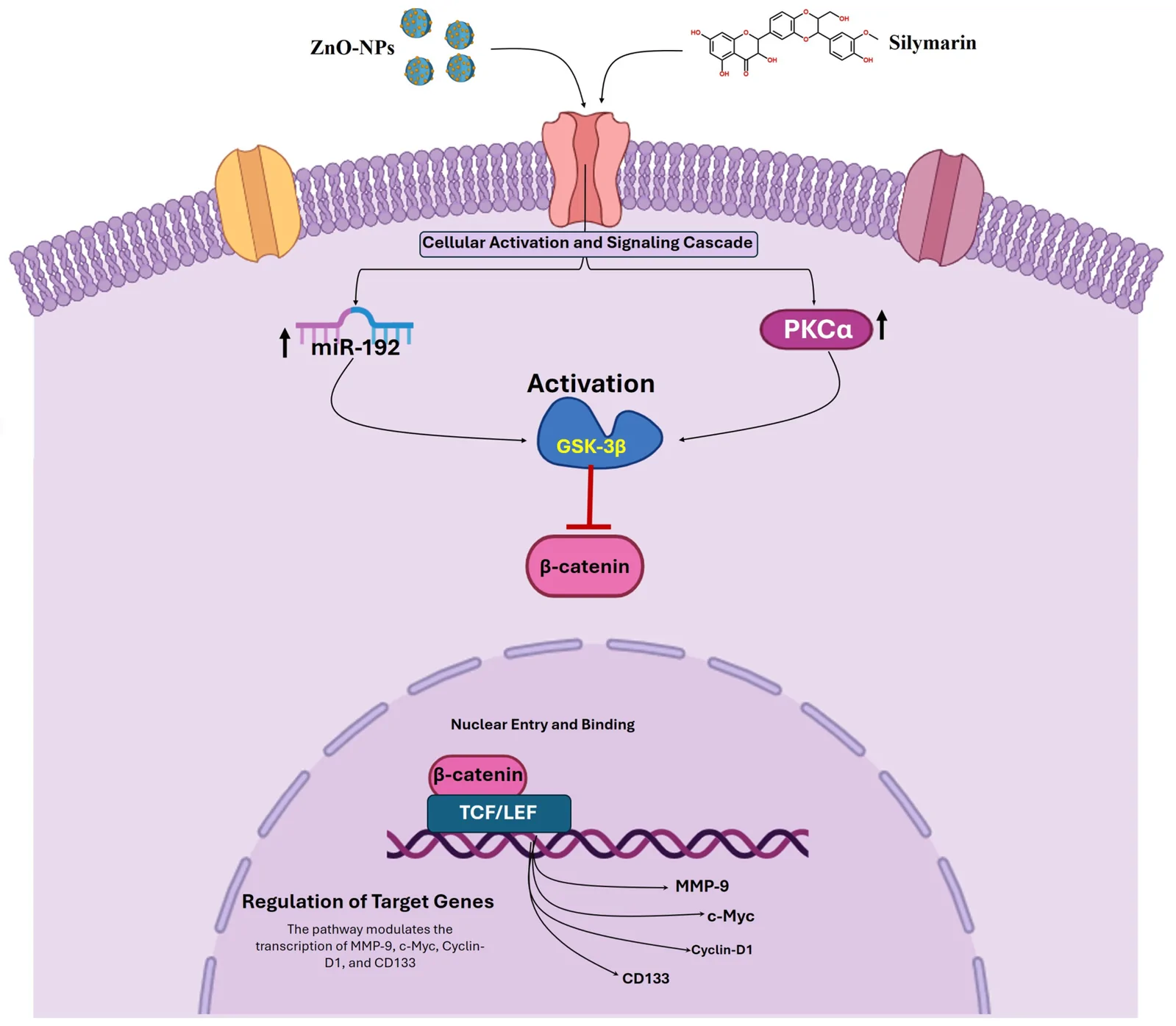
Proposed mechanism of silymarin and ZnO-NP action. Silymarin and ZnO-NPs attenuate DMH-induced colon cancer by modulating miR-192 and PCKα, which, in turn, inhibit wnt/β-catenin pathways and their downstream genes that are normally essential for tumor growth, proliferation, metastasis, and cancer stemness.

In conclusion, the combined administration of Sily and ZnO-NPs produced greater antitumor effects in DMH-induced colon cancer than either treatment alone. This improvement was accompanied by a better oxidative status, restoration of miR-192 and PKCα expression, reduced inhibitory phosphorylation of GSK-3β, and lower β-catenin expression. The combined treatment also decreased cyclin D1, c-Myc, MMP-9, and CD133 expression and improved colonic histopathological features. These findings suggest that Sily and ZnO-NPs may exert complementary effects on several processes involved in colon carcinogenesis. The changes observed in miR-192/PKCα and GSK-3β/β-catenin signaling may contribute to these effects, although their direct mechanistic involvement remains to be established. Overall, the present findings support further preclinical investigation of this combination. Additional studies are needed to evaluate its pharmacokinetics, biodistribution, long-term safety, and efficacy in other experimental models and with clinically relevant routes of administration before its translational potential can be determined (Figure 16).

## 6. Limitations and Future Directions

While this study provides comprehensive biochemical, molecular, and histological evidence regarding the therapeutic efficacy of the Sily/ZnO-NP combination in DMH-induced colon cancer, certain methodological limitations should be acknowledged to guide future investigations. First, comprehensive systemic safety evaluations, including hemogram profiles, serum biochemistry (ALT, AST, ALP, creatinine, and urea), and multi-organ histopathology, along with Zn^2+^ dissolution kinetics, biological media stability, and endotoxin testing, should be incorporated to fully establish the clinical safety and systemic toxicity profile of this treatment. Second, because whole-tissue homogenates capture net redox status, subsequent studies utilizing tumor-cell-specific ROS probes, immunofluorescence-based oxidative markers, or spatially resolved analyses will be valuable for distinguishing localized pro-oxidant anticancer dynamics from broader tissue-level redox adaptations. Third, while in vivo molecular changes and docking predictions indicate significant pathway modulation, direct target-engagement approaches such as purified GSK3β enzymatic activity assays, subcellular fractionation or immunofluorescence for nuclear β-catenin localization, and assessments of canonical Wnt targets (e.g., Axin2 and Lgr5) are required to confirm direct causality. Fourth, future studies employing miR-192 mimics/inhibitors, PKCα modulation, TCF/LEF reporter assays, and in vitro cell-line interaction matrices (using Bliss or Loewe models) will further refine our understanding of the precise molecular mechanisms and synergistic interactions driving this combination therapy. Finally, while tumor incidence and multiplicity were successfully quantified alongside blinded histopathological assessments, early aberrant crypt foci (ACF) tracking was beyond the scope of this advanced post-induction therapeutic model; thus, future prevention-focused protocols will incorporate early ACF monitoring alongside macroscopic endpoints.

## Figures and Tables

**Table 1:** Primer sequences for RT-PCR.

Gene of Interest		Primer Sequence	Reference Sequence	Product Size
β-actin	F	5`-CTGTGTGGATTGGTGGCTCT-3`	NM_031144.3	134
	R	5`-AGCTCAGTAACAGTCCGCC-3`		
PKCα	F	5`-TGAATCCTCAGTGGAATGAGT-3`	NM_001429972	325
	R	5`-GGTTGCTTTCTGTCTTCTGAA-3`		

**Table 2:** Effect of Sily, ZnO-NPs, and Sily/ZnO-NPs on the tumor incidence and tumor multiplicity in DMH-induced colon cancer in rats.

Groups	Tumor Incidence	Tumor Multiplicity
Control	0%	0
CC	100%	4 ± 0.3651
Sily	83.3%	1.5 ± 0.4282
ZnO-NPs	83.3%	1.167 ± 0.3073
Sily/ZnO-NPs	33.3%	0.3333 ± 0.2108

**Table 3:** Binding affinities and molecular interactions of Sily and ZnO-NPs with GSK-3β.

Molecular Target and PDB Code	Ligand	Hydrogen-Bond Analysis		Amino Acids Involved in Lipophilic Analysis	Binding Affinity (kcal/mol)
		Amino Acid Involved in H Bond	Distance (Å)		
GSK-3β (6Y9S)	Sily	ARG141	3.1	ALA83, ILE62, LEU132, VAL70, PHE67, VAL135, LEU188, VAL110, CYS199, PHE201, and MET101	−9.5
		LYS85	2.1		
		CYS199	3.9		
	ZnO-NPs	LYS183	2.9	LEU188, VAL135, ALA83, ILE62, VAL70, PHE67, GLY63, CYS199, GLY65, THR138, SER66, and TYR134	−7.8
			2.8		
		ASN186	3.6		

## Data Availability

The original contributions presented in this study are included in the article. Further inquiries can be directed to the corresponding author.

## Supplementary Materials

The following are available online at https://www.mdpi.com/article/10.3390/biom16091374/s1, File S1: original Western Blot images.
