# Supplementary material for: Silymarin and Zinc Oxide Nanoparticles Attenuate Experimental Colon Cancer in Rats Through Crosstalk of miR-192 and β-Catenin Pathways
Source: Biomolecules. 2026 Sep 21;16(9):1374. doi: 10.3390/biom16091374 (PMC13604585; doi:10.3390/biom16091374)
Supplement: Supplementary file 1 [file biomolecules-16-01374-s001.zip › File S1 original Western Blot images.pdf]

β-actin (42 kDa)

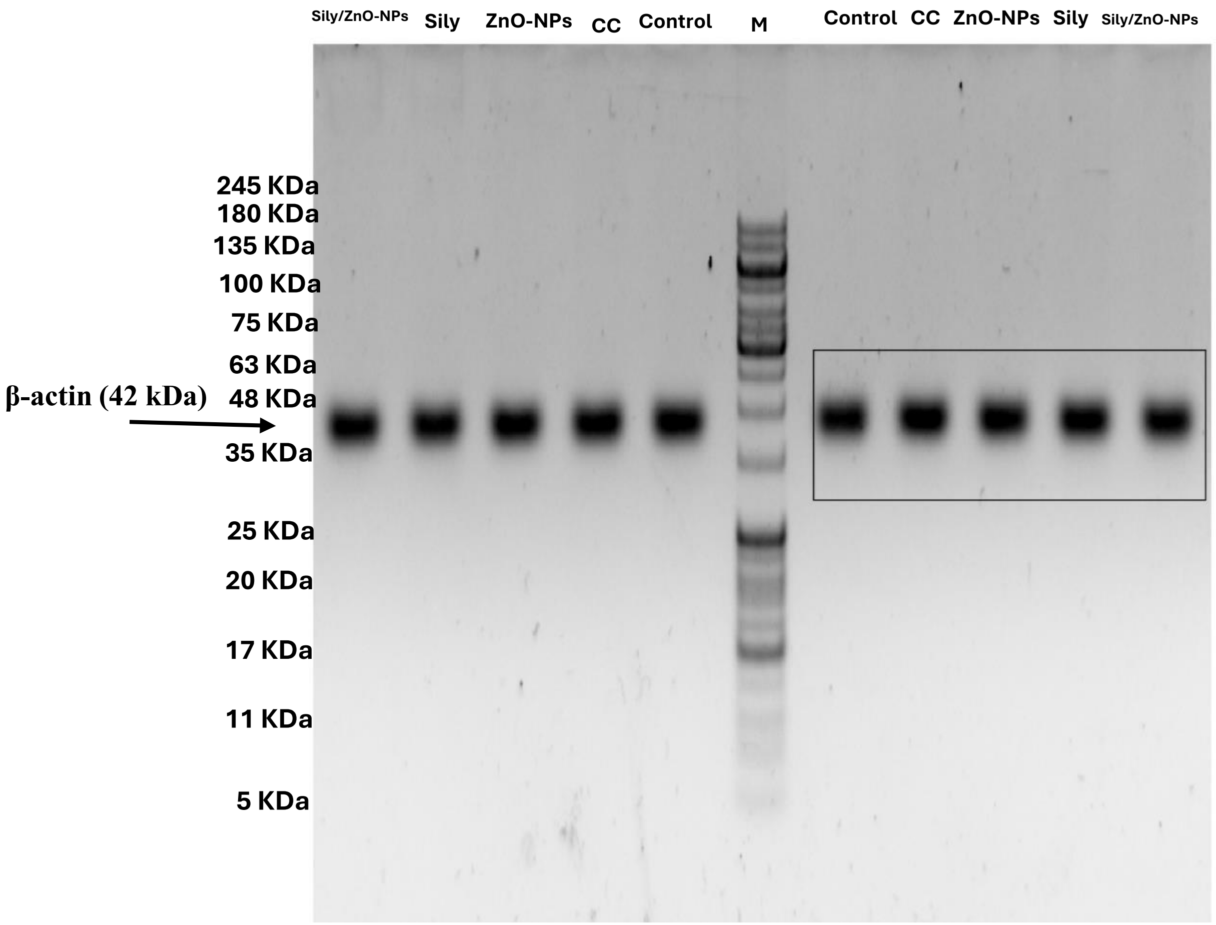

M: Pre-stained protein molecular weight marker

Box indicates the cropped region shown in the manuscript figure.

t-GSK-3β (~46–47 kDa)

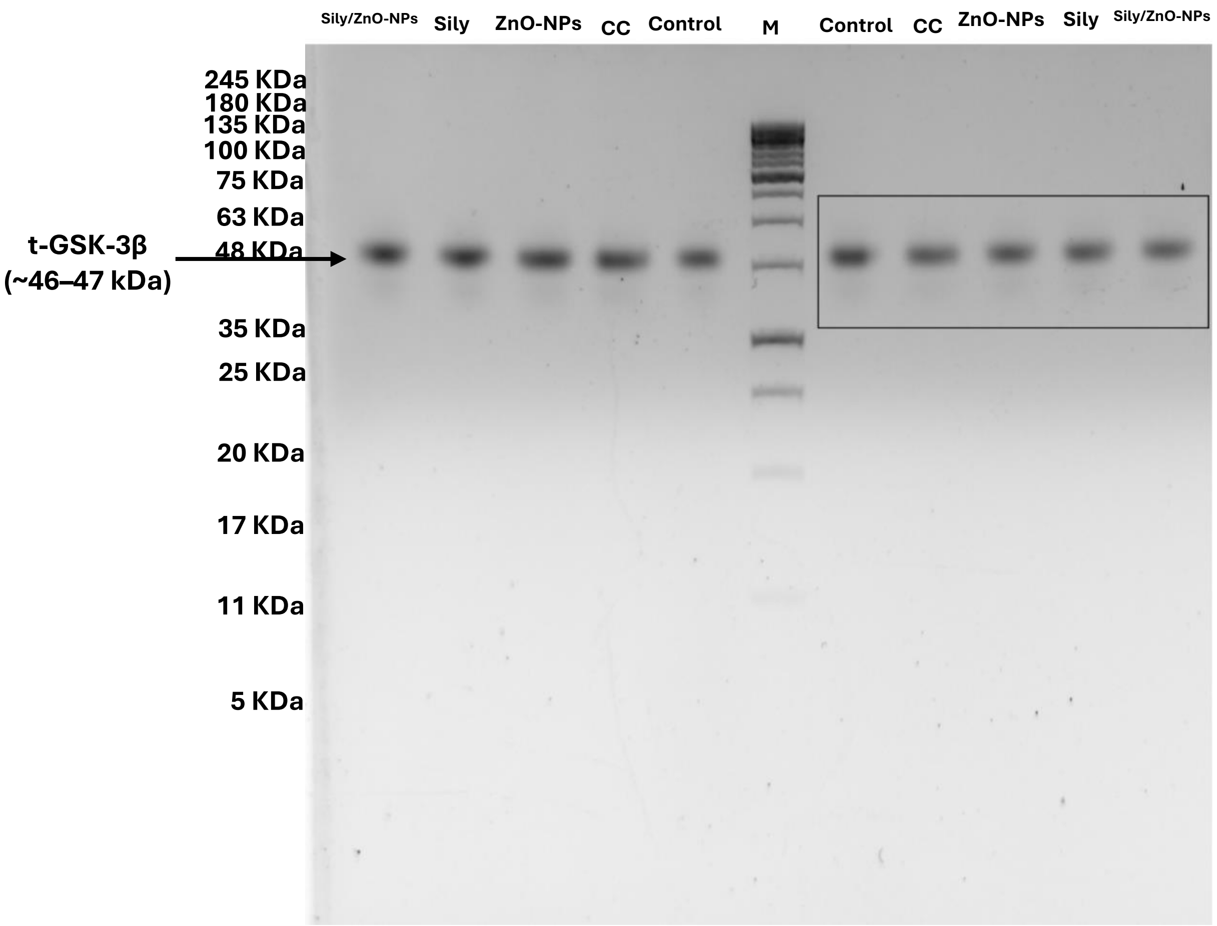

M: Pre-stained protein molecular weight marker

Box indicates the cropped region shown in the manuscript figure.

p-GSK-3β(~47 kDa)

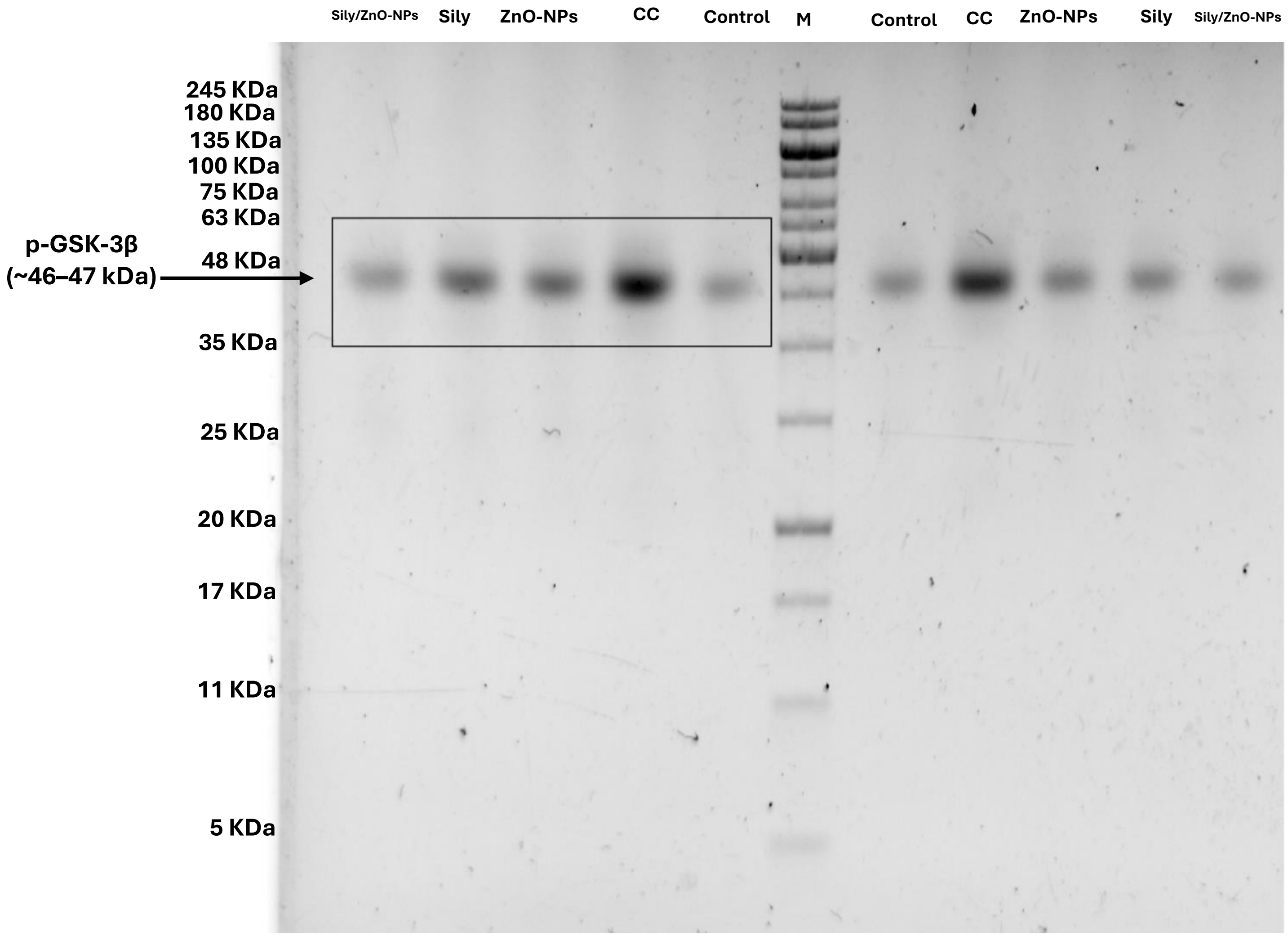

M: Pre-stained protein molecular weight marker

Box indicates the cropped region shown in the manuscript figure.
All samples and controls were run on the same gel and blot.
